# Tunable Hybrid Matrices Drive Epithelial Morphogenesis and YAP Translocation

**DOI:** 10.1002/advs.202003380

**Published:** 2020-12-11

**Authors:** Ying Zhang, Mirjam M. P. Zegers, Anika Nagelkerke, Alan E. Rowan, Paul N. Span, Paul H. J. Kouwer

**Affiliations:** ^1^ Institute for Molecules and Materials Radboud University Heyendaalseweg 135 Nijmegen 6525 AJ The Netherlands; ^2^ Radiotherapy and OncoImmunology Laboratory, Department of Radiation Oncology Radboud University Medical Center Geert Grooteplein 32 Nijmegen 6525 GA The Netherlands; ^3^ Department of Cell Biology, Radboud Institute for Molecular Sciences Radboud University Medical Center Geert Grooteplein 28 Nijmegen 6525 GA The Netherlands; ^4^ Present address: Pharmaceutical Analysis, Groningen Research Institute of Pharmacy University of Groningen P.O. Box 196, XB20 Groningen 9700 AD The Netherlands; ^5^ Australian Institute for Bioengineering and Nanotechnology (AIBN) The University of Queensland Brisbane QLD 4072 Australia

**Keywords:** 2D and 3D matrices, epithelial morphogenesis, hybrid hydrogels, MDCK cells, polyisocyanides, YAP

## Abstract

Morphogenesis is a tightly‐regulated developmental process by which tissues acquire the morphology that is critical to their function. For example, epithelial cells exhibit different 2D and 3D morphologies, induced by distinct biochemical and biophysical cues from their environment. In this work, novel hybrid matrices composed of a Matrigel and synthetic oligo(ethylene glycol)‐grafted polyisocyanides (PICs) hydrogels are used to form a highly tailorable environment. Through precise control of the stiffness and cell‐matrix interactions, while keeping other properties constant, a broad range of morphologies induced in Madin‐Darby Canine Kidney (MDCK) cells is observed. At relatively low matrix stiffness, a large morphological shift from round hollow cysts to 2D monolayers is observed, without concomitant translocation of the mechanotransduction protein Yes‐associated protein (YAP). At higher stiffness levels and enhanced cell‐matrix interactions, tuned by controlling the adhesive peptide density on PIC, the hybrid hydrogels induce a flattened cell morphology with simultaneous YAP translocation, suggesting activation. In 3D cultures, the latter matrices lead to the formation of tubular structures. Thus, mixed synthetic and natural gels, such as the hybrids presented here, are ideal platforms to dissect how external physical factors can be used to regulate morphogenesis in MDCK model system, and in the future, in more complex environments.

## Graphical abstract

The cellular environment plays a key role in determining tissue morphogenesis. This work uses hybrid matrices, composed of synthetic polyisocyanides (PICs) and Matrigel, that allows for precise control of matrix stiffness and cell‐matrix interactions. Variations in hybrid matrices induce a full spectrum of morphologies in Madin‐Darby canine kidney (MDCK) cells, and includes both YAP‐dependent and ‐independent processes.

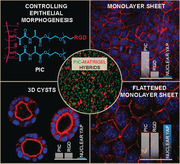

## 1 Introduction

Epithelial tissues are widely found in our bodies, for instance as extended monolayer sheets (2‐dimensional, 2D) that line all surfaces of the body^[^ ^1^ ^]^ or as spherical or tubular structures surrounding a central lumen (3‐dimensional, 3D), participating in diverse physiological functions, including protection, digestion, reproduction, excretion, and hormone secretion. Epithelial cells are polarized, generating apical and basal surfaces.^[^ ^2^ ^]^ The basal surface interacts with the supporting basement membrane of 2D cell sheets and 3D structures.^[^ ^3^ ^]^ The broad range of epithelial morphologies found in vivo requires excellent control of the processes that drive morphogenesis.^[^ ^4^, ^5^ ^]^ In vitro, however, epithelial morphogenesis is strongly dependent on experimental conditions: cells typically grow into monolayer sheets when they are seeded on flat 2D substrates, and into well‐organized cysts when cultured in a 3D matrix.^[^ ^1^ ^]^ Clearly, 2D and 3D culture conditions are principally different, and at this stage it remains unclear how external cues contribute to the shift from 2D monolayer sheets and 3D cysts.

In vivo tissue morphogenesis is driven by forces generated by actin‐myosin networks and transmitted through the cytoskeleton.^[^ ^6^, ^7^ ^]^ Consequently, the mechanical properties of the surrounding matrix, amongst other (biochemical) cues, have been found to impact morphogenesis.^[^ ^8^, ^9^, ^10^ ^]^ Matrix mechanical properties are transmitted through cell matrix adhesions to the cytoskeleton, where actin and myosin play key roles. In the past decade, Yes‐associated protein (YAP) was identified to play an important role in mechanotransduction:^[^ ^11^ ^]^ upon activation, YAP shuttles from the cytoplasm to the nucleus, where it regulates transcription, ultimately driving proliferation, apoptosis and morphogenesis.^[^ ^12^, ^13^ ^]^ Numerous researchers have demonstrated that experimental manipulation of the matrix architecture and/or mechanical properties affect YAP.^[^ ^14^, ^15^ ^]^ Recent work,^[^ ^16^ ^]^ however shows that YAP activity may also depend on the culture dimensionality (2D versus 3D), independent of matrix stiffness. In fact, the role of YAP on morphogenesis and, by extension, many other processes such as tumorigenesis and cancer progression, remain poorly understood.

A challenge in the field is to find well‐defined matrices and substrates with tunable properties that are able to provide an environment that supports the formation of different cell morphologies. Currently used artificial extracellular matrices (ECMs) can be crudely categorized into animal‐derived and synthetic materials. Both provide advantages, but certainly also disadvantages that complicate a systematic investigation of the effects of biochemical and biophysical cues on cell behavior. Animal‐derived matrices provide many signals and it is difficult to tune a desired parameter (such as stiffness) without affecting all other signals. Such properties can be engineered precisely (and independently) in synthetic matrices, but they typically lack spatiotemporal variations in chemical and physical properties while interacting with cells. Hybrid hydrogels based on synthetic polymers and natural polymers form an excellent approach to overcome the disadvantages, and allow unique insights in cell behavior by precise control over the physiological properties of the microenvironment.^[^ ^16^, ^17^, ^18^ ^]^

A relatively new class of synthetic materials in the field comprises tri(ethylene glycol)‐grafted polyisocyanide (PIC) hydrogels. PIC gel formation is thermally induced by heating beyond the gelation temperature (*T*_gel_ ≈ 15 °C), resulting in a transparent elastic gel. The thermoresponsive nature is completely reversible. As a synthetic material, gel properties, such as mechanical properties and architecture are readily tailored, for instance by changing the concentration, polymer molecular weight (length), by controlling external factors, such as temperature and ionic strength, and by the addition of other components to make hybrid structures.^[^ ^19^, ^20^, ^21^, ^22^, ^23^ ^]^ Like collagen and fibrin, PIC gels form a fibrous and porous network, already at low concentrations.^[^ ^19^ ^]^ The architecture is highly heterogeneous and pore sizes at the micron length scale are (weakly) dependent on the polymer concentration.^[^ ^24^ ^]^ The fibrous network architecture gives rise to interesting mechanical properties, including a strong strain stiffening response, where the stiffness of the gel can increase 100‐fold after a stress is applied to the gel.^[^ ^25^ ^]^ This instantaneous and reversible response is also found in biogels based on fibrin and collagen,^[^ ^26^ ^]^ but is rarely observed in synthetic gels. Recent work indicates the importance of strain‐stiffening for cell behavior^[^ ^27^, ^28^, ^29^, ^30^ ^]^ and the suitability of PIC gels as a synthetic matrix material.^[^ ^31^, ^32^ ^]^ Early experiments in vivo demonstrate the biocompatibility of PIC gels and the lack of an immunogenic response.^[^ ^33^, ^34^ ^]^ For biological applications, PIC polymers are routinely functionalized for cell adhesion through the well‐known adhesive peptide, Gly‐Arg‐Gly‐Asp‐Ser (abbreviated to RGD), a motif found in many ECM proteins, including collagen, fibronectin, tenascin and vitronectin and binds several integrins.^[^ ^35^, ^36^ ^]^

Here, we take advantage of the biomimetic and tailorable properties of PIC gels to form novel hybrid gels with animal‐derived Matrigel to study morphogenesis in the epithelial MDCK cell line. MDCK cells have retained many features of polarized kidney tubules, making them an excellent model to study epithelial morphogenesis.^[^ ^37^, ^38^ ^]^ Depending on the ECM conditions, these cells grow as contact‐inhibited monolayers, cysts, or form branching tubules, which turned MDCK cells into a well‐established model for delineating the mechanisms involved in ECM‐dependent epithelial polarization and tubulogenesis.^[^ ^3^ ^]^ In the hybrid hydrogels‐based ECMs, we are able to precisely control the mechanical properties and the density of the ligands for mechanotransduction via the PIC gel, while Matrigel likely provides interaction with ECM components, such as laminin, which are crucial to control morphogenesis.^[^ ^39^, ^40^, ^41^ ^]^ Our main research question is: can we control MDCK morphogenesis simply by tuning matrix characteristics and, if this is the case, are non‐muscle myosin II, actin and YAP involved in the mechanotransduction process?

## 2 Results

### 2.1 Material Preparation and Characterization

**Figure 1:**
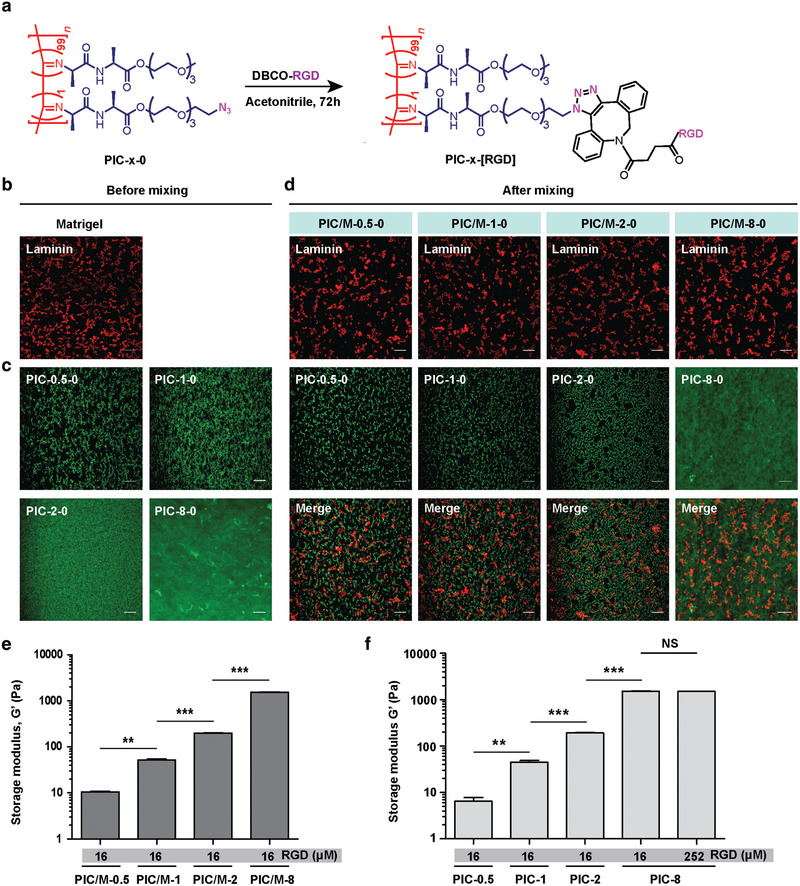
Materials used in this work. a) RGD conjugation of PIC through the efficient strain‐promoted azide‐alkyne cycloaddition reaction. The full structure is given in Figure S1, Supporting Information. b) Representative immunofluorescence image of 4.4 mg mL^−1^ Matrigel, stained with laminin‐111. c) Representative confocal images of PIC hydrogels with Cy3 conjugated (different concentrations). d) Representative immunofluorescence images of PIC/M hydrogels: laminin‐111 signal from Matrigel (top); Cy3 signal of PIC (middle) and merge (bottom). All images are representative of *n* = 3 independent experiments. Scale bar in panels (b–d): 20 µm. e) Storage modulus *G′* of PIC/M hydrogels with different PIC concentrations. f) Storage modulus *G′* of PIC gels (with RGD) showing the impact of the gel concentration. NS = not significant, **p < 0.01, ***p < 0.001, one‐way ANOVA followed by Tukey's multiple comparisons test. The given values of *G′* are the averages of at least three independent measurements. Bars represent mean SEM.

We prepared matrices based on hybrid hydrogels of Matrigel and the synthetic PIC gel in different ratios and specifications (we use notation PIC/M to denote the hybrid gels). Matrigel is a commercially available animal‐derived matrix that is widely used for in vitro cell culture studies and was used as received. For the PIC/M hydrogels in this work, the Matrigel concentration is always constant with a protein concentration 4.4 mg mL^−1^. PIC gels are synthetic materials that are prepared in our laboratories. The synthesis of the PIC and its conjugation is summarized in Figure S1, Supporting Information, following previously reported procedures.^[^ ^29^, ^30^ ^]^ In short, the polymer was prepared from a mixture of monomer containing 1% azide‐appended monomer for conjugation, and a nickel catalyst in a monomer:catalyst ratio of 2000:1, which is the default setup in our lab. The molecular weight of PIC was measured with viscometry^[^ ^42^ ^]^ and yielded *M*_v_ = 419 kDa, which corresponds to an average degree of polymerization *n* = 1325 and polymer contour length *L* = 166 nm.^[^ ^20^ ^]^ The cell‐binding RGD‐based peptide was conjugated to the azide groups using the highly efficient strain‐promoted azide‐alkyne cycloaddition reaction (**Figure** **1**a; Figure S1, Supporting Information).^[^ ^28^, ^43^ ^]^ Gels with reduced RGD concentrations were prepared by mixing RGD‐conjugated PIC with the PIC azide precursor in the appropriate ratios. PIC bundle formation ensures a homogeneous presentation of cell binding sites in the gel.

To form a gel, a cold PIC stock solution (16 mg mL^−1^) was prepared by dissolving the sterilized (UV treatment, 5 min) solid polymer in the desired amount of chilled sterile HBSS. The PIC solution(s) was mixed with pre‐cooled Matrigel and additional cold HBSS was added when necessary. Heating the solution beyond the PIC gelation temperature *T*_gel_ ≈ 15 °C resulted in instantaneous (and reversible) gel formation. In this manuscript, we vary PIC concentrations and RGD densities in both PIC/M hydrogels and PIC gels. For clarity, we will use the notation PIC‐*c*‐[RGD] and PIC/M‐*c*‐[RGD] where *c* is the PIC concentration (in mg mL^−1^) and [RGD] is the concentration adhesive peptide (in µm).

### 2.2 PIC‐Matrigel Hybrid Hydrogel Analysis

When preparing the PIC/M hydrogels, we first confirmed that gel formation of both components was not mutually influenced by assessing the gel morphology (microscopic) and the mechanical properties (macroscopic). For the former, we used confocal fluorescence microscopy, for which we immunostained laminin‐111, a major component of Matrigel (Figure 1b) and conjugated Cy3 to the PIC chains (Figure 1c). The PIC/M gels were prepared by mixing both components at low temperatures and warming them to 37 °C. Fluorescence microscopy of the PIC/M hydrogels (Figure 1d) shows that laminin‐111 is evenly distributed in all samples (independent of the PIC concentration), indicative of a homogeneous mixing of the Matrigel components; confocal stacks indicate that both components are tightly mixed (Figure S2, Movies S1 and S2, Supporting Information). The PIC component becomes increasingly dense as its concentration increases as is observed from the fluorescence images (Figure 1b–d). Recent work shows that the micron‐scale pore size in PIC gels reduces slightly with increasing polymer concentration.^[^ ^24^ ^]^ As for the mechanical properties of the PIC/M hydrogels, we observed relatively soft gels with (shear) storage moduli *G*′ between 10 and1500 Pa for PIC gel concentrations *c* = 0.5–8 mg mL^−1^ (Figure 1e; S3a, Supporting Information). In the PIC/M hybrids, the stiffness contribution of 4.4 mg mL^−1^ Matrigel, which forms a very soft hydrogel on its own (*G*′ ≈ 1 Pa), is very small (Figure S3a, Supporting Information), which means that the stiffness is dominated by the concentration of PIC (Figure 1e,f; Figure S3b, Supporting Information), and that observed mechanical effects primarily originate from the PIC network.^[^ ^44^ ^]^ We note that the gels are mostly elastic (with *G*′ ≫ *G*″) and that the strain stiffening behavior, although not a focus of this work, is largely maintained in the hybrids (Figures S4 and S5, Supporting Information). With a constant Matrigel concentration and a largely unperturbed Matrigel architecture, the biochemical cues are comparable between samples, while mechanical cues through matrix stiffness and cell adhesion to the load‐carrying PIC network can be tuned with the PIC concentration and the RGD concentration. This tunability provides a unique opportunity to study the role of external mechanical responses and RGD‐mediated cell‐ECM interactions in directing epithelial morphogenesis.

### 2.3 MDCK Behavior on Matrigel or PIC Hydrogels

The epithelial cell line MDCK is a well‐established model for epithelial morphogenesis and apical‐basal cell polarization. On stiff adhesive 2D surfaces they form polarized monolayers with apical domains facing the medium, whereas in biological 3D matrices they form polarized hollow cysts with apical surfaces facing the cyst lumen. Before studying morphogenesis on and in the PIC‐Matrigel hybrid gels, we studied the effects of the Matrigel and PIC gels separately in 2D cultures.

MDCK cells (20 000 mL^−1^) were seeded on top of solidified Matrigel (4.4, 6.2 and 8.8 mg mL^−1^). After 4 days in culture, the cells developed into round hollow cysts, regardless of the Matrigel concentration (Figure S6a, Supporting Information), fully in line with earlier work.^[^ ^45^ ^]^ For component‐rich matrices, such as Matrigel, changing the concentration does not only change the stiffness, but also the concentration of all bioactive factors in the matrix, which makes it challenging to draw unambiguous conclusions.

At the opposite side of the spectrum are the minimal synthetic matrices such as PIC, that only provide a mechanically controlled environment—with or without cell‐matrix adhesion—but lack any other biochemical cues or factors. After culturing for 4 days, MDCK cells seeded on top of different concentration solidified PIC gels all showed the formation of big cell clusters and clumps, independent of the PIC concentration (Figure S6b, Supporting Information). The cell clusters and clumps showed low adhesion to the substrate, that is, they were washed off easily. The apical surface membrane marker gp135 is localized in the outer surface of the clumps (Figure S6c, Supporting Information), instead of its normal luminal localization, similar to other (3D) experiments in which cell‐matrix adhesion was inhibited.^[^ ^46^ ^]^ Thus, without RGD conjugated to the polymer, the MDCK cells are unable to interact with the matrix and cannot sense its physical properties. After the introduction of low concentrations of cell‐adhesive peptides (PIC‐0.5‐16, PIC‐1‐16, PIC‐2‐16, and PIC‐8‐16), we observe different cell behavior: in these cases, 2D monolayer sheets are formed with apical domain facing the medium on the gel substrate for all PIC concentrations (Figure S6d,e, Supporting Information). Note that an overview of MDCK cell behavior on PIC gels, PIC/M hydrogels and controls is given in Table S1, Supporting Information.

### 2.4 MDCK Cell Morphogenesis in PIC‐Matrigel Hybrid Matrices

In the PIC/M hydrogels, cells can potentially interact with RGD conjugated to the PIC and with various matrix components in Matrigel. To study if the introduction of RGD on the PIC network is necessary at all, we first prepared hybrids of Matrigel and PIC without RGD. In these samples, all cell‐matrix adhesion is through Matrigel components.

**Figure 2:**
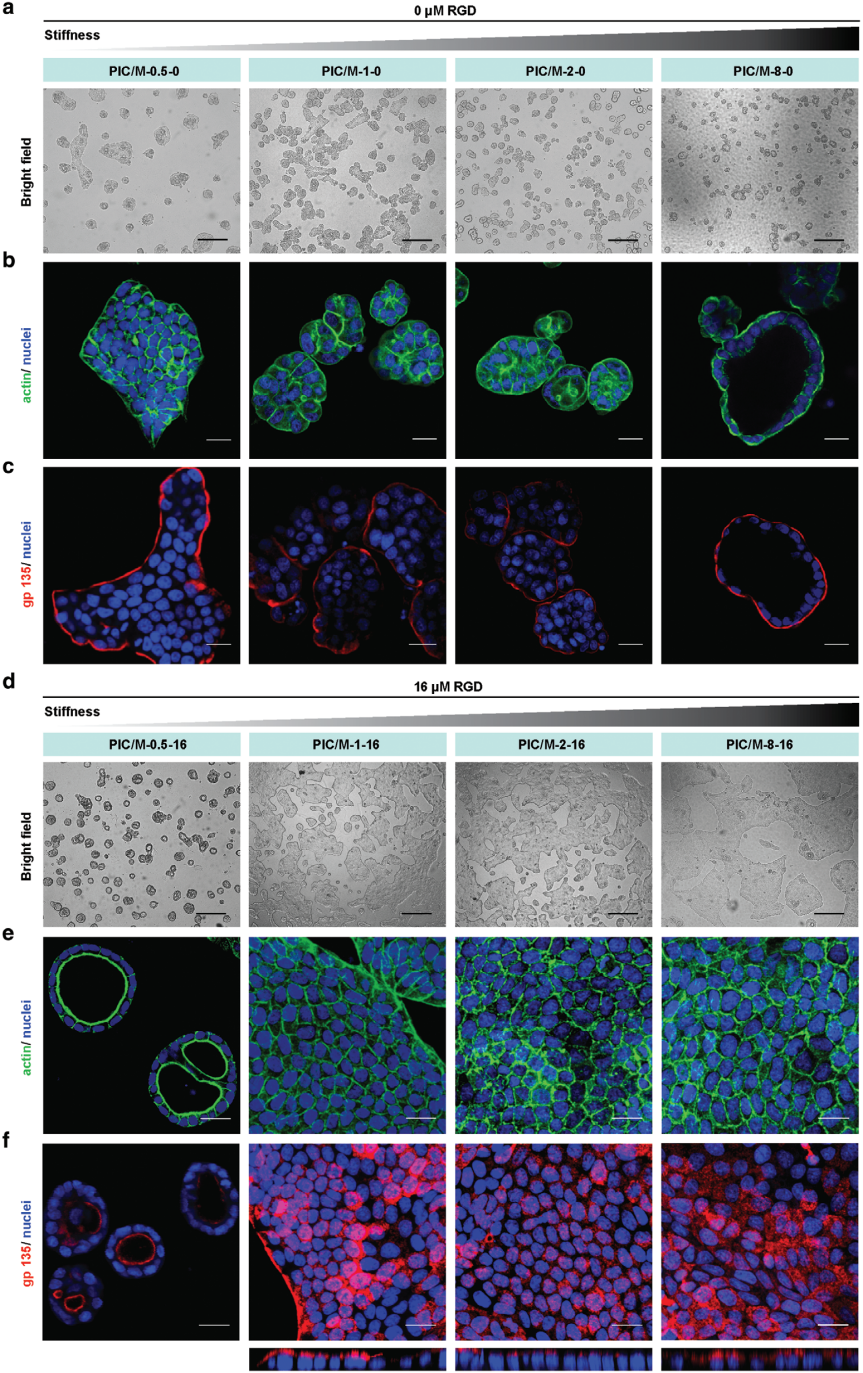
2D culture of MDCK cells on substrate of different stiffness. a–c) Representative bright field images (a), actin staining (b), and gp135 immunofluorescence staining (c) of MDCK cells on PIC/M hydrogels without RGD conjugated to the polymers. (d–f) Representative bright field images (d), actin staining (e) and gp135 immunofluorescence images (f) of MDCK cells on PIC/M hydrogels with 16 µm RGD conjugated to PIC. Bottom panel: confocal z‐stack. Phalloidin: green; gp135: red; nuclei: blue. All images are representative of *n* = 3 independent biological experiments. Bright field scale bar: 50 µm. Fluorescence scale bar: 20 µm.

We seeded MDCK cells on PIC/M hydrogels with the same Matrigel concentration (4.4 mg mL^−1^) and varying PIC concentrations (0.5, 1, 2, 8 mg mL^−1^), first without RGD attached to the PIC (preventing direct mechanical cell‐PIC interaction), and analyzed the cell morphologies after 4 days in culture (**Figure** **2**). Cells on soft and intermediate matrices PIC/M‐0.5‐0 (i.e., 0.5 mg mL^−1^ PIC and no RGD), PIC/M‐1‐0, and PIC/M‐2‐0 generated round (but not hollow) clusters or formed small monolayer clusters (Figure 2a,b). Polarization analysis by gp135 immunostaining (Figure 2c) shows that the cell structures display an apical layer facing the medium. On the stiffest PIC/M‐8‐0, hollow clusters were formed, also with the apical side facing the medium and not the lumen, which means with an inverted polarization compared to the in vivo‐like situation.

The polarity of epithelial cells is strongly influenced by neighboring cells and their extracellular environment.^[^ ^47^ ^]^ Despite the presence of Matrigel, we still find disorderly polarization in PIC/M hybrid matrices of RGD‐deficient PIC. To improve cell‐matrix interactions in the PIC/M hydrogels, we introduced minimal amounts of cell‐adhesive peptide to the PIC polymer: a final concentration of 16 µm RGD in all hybrids. Note that other work studying the effect of RGD typically applies much higher concentrations (100–2000 µm).^[^ ^48^ ^]^ MDCK cells cultured on these matrices showed well‐defined morphologies (Figure 2d–f): at the lowest PIC density (PIC/M‐0.5‐16), we observed cyst formation with the correct polarization, that is, the apical side facing the lumen. Subsequent staining for laminin‐111, a major Matrigel component, showed the formation of a basement membrane (Figure S7, Supporting Information), which suggests active remodeling of the Matrigel matrix by the cell.^[^ ^49^ ^]^ All stiffer gels gave rise to the formation of extended 2D cell sheets (monolayers) with their apical side facing the medium. Interestingly, introducing very small amounts of adhesion sites on the synthetic component of the hybrid matrix allowed the successful construction of different epithelial morphologies with in vivo‐type polarities. The data also showed that relatively small changes in the physical cues of the matrix, not only the stiffness but also the presentation of adhesive peptides had major impact on morphogenesis. We note that proliferation assays confirm that the PIC/M substrates support MDCK cells growth (Figure S8, Supporting Information) in line with earlier work using PIC‐based cell culture matrices.^[^ ^29^, ^30^, ^31^ ^]^

**Figure 3:**
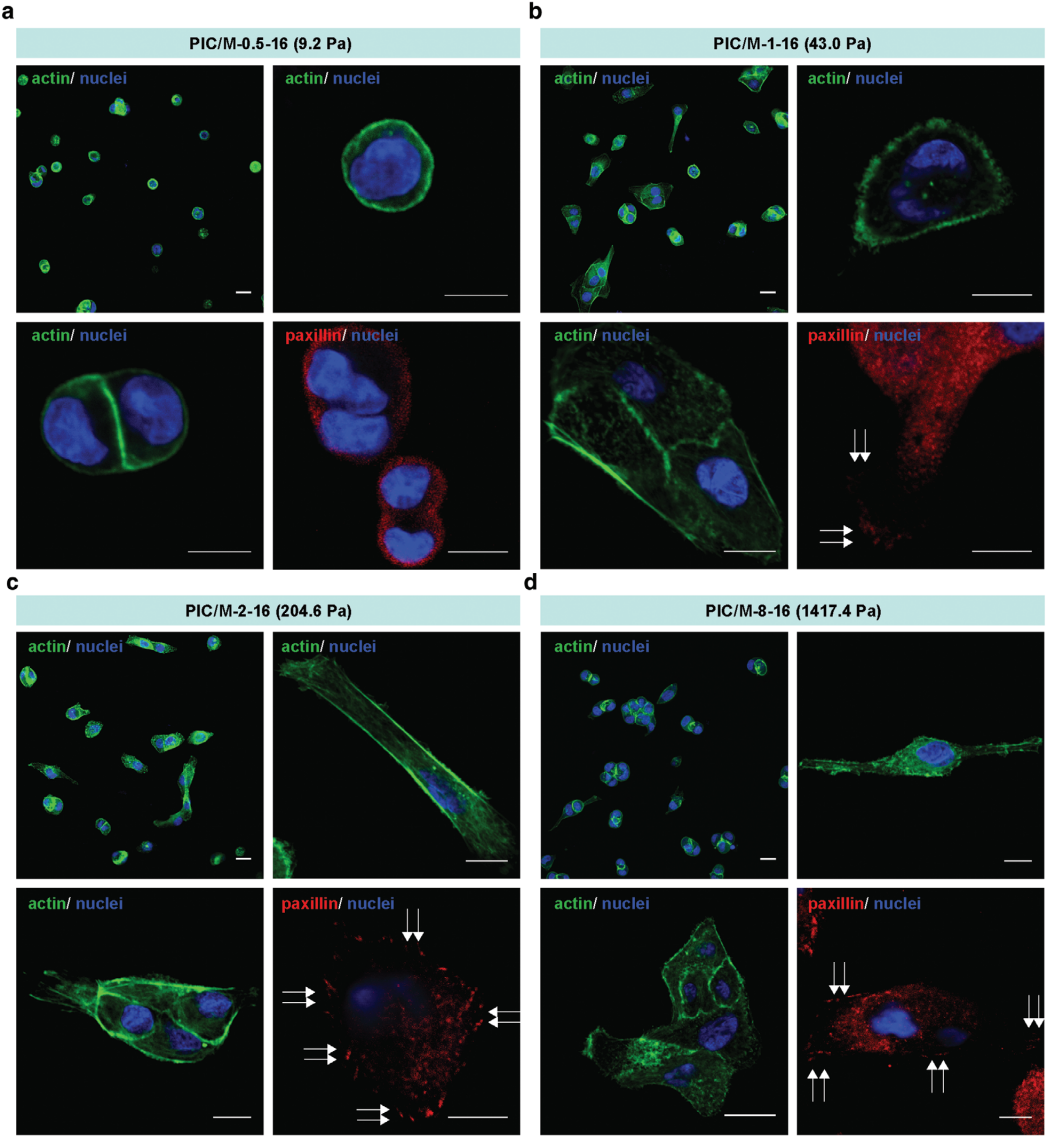
MDCK cell‐ECM interaction analysis. a–d) Representative actin and paxillin staining images of MDCK cells on PIC/M‐0.5 (a), PIC/M‐1 (b), PIC/M‐2 (c) and PIC/M‐8 (d) recorded 5 h after cell seeding. Arrows indicate accumulated paxillin. All images are representative of *n* = 3 independent biological experiments. Scale bar: 10 µm.

The shift from 3D cysts to monolayers we show here is the result of changes in substrate properties, most likely sensed by cells through RGD‐integrin‐based cell‐ECM interactions that result in downstream effects for the actin cytoskeleton. To better understand how the substrate properties influence processes like actomyosin contraction and focal adhesion (FA) formation, we studied the cells at early stages, that is, 5 h after plating. F‐actin was stained to observe cell morphologies, and paxillin to visualize FAs (**Figure** **3**). Cells on the soft PIC/M hydrogels with RGD (PIC/M‐0.5‐16) proliferated, but did not show signs of stress fiber formation or spreading, nor focal accumulation of paxillin around the edges; all indicative of the absence of FAs (Figure 3a). On the stiffer substrates that induce 2D cell sheet formation, the cells clearly spread after 5 h and paxillin is found in the cell periphery (Figure 3b–d). On these stiffer substrates we also see that after cell adherence the cytoskeleton remodels, which involves actin and non‐muscle myosin II activity and the formation of stress fibers and FAs. The latter improves the interaction with the substrate, which leads to an effective mechanical feedback that promotes cell spreading. In short, even in this soft matrix regime, we clearly observe differential responses to the matrix stiffness. On the softest material, lack of a mechanical response of the substrate prevents FA formation, permitting cells to freely grow and remodel their environment, ultimately promoting cyst formation. On slightly stiffer matrices, cells spread and form FAs that bind to the substrate, which promotes the formation of 2D monolayer sheets.

The fact that matrix stiffness controls morphogenesis is widely accepted,^[^ ^8^, ^50^ ^]^ but in this case, the switch from cyst to monolayer sheets was already seen at a very low stiffness *G*′ < 40 Pa. Also, as YAP has been identified as one of the key players in mechanotransduction, we immunostained for YAP protein and analyzed its localization. To our surprise, we found that in all samples, YAP resides in the cytoplasm with no obvious translocation to the nucleus (**Figure** **4**a; Figure S9, Supporting Information). In other words, the morphological shift of MDCK cells from 3D cysts to 2D monolayers, which is triggered by substrate stiffness, is not accompanied by a nuclear translocation of YAP.

Figure 4MDCK mechanical sensing analysis. a) Representative immunofluorescence staining images of YAP and nuclei in MDCK cells on PIC/M hydrogels. Note that in all conditions YAP is cytoplasmic. As a positive control, we observe nuclear YAP at higher RGD concentration (Figure S10, Supporting Information). b) Representative actin staining images of MDCK cells on PIC/M hydrogels after cell seeding for 5 h. c,d) Representative actin staining images of MDCK cells on PIC/M hydrogels treated with cytochalasin D (c) and blebbistatin (d) after cell seeding for 5 h. Inhibitors were added on 0 h. e) Representative actin staining images of MDCK cells on PIC/M hydrogels after cell seeding for 96 h. f,g) Representative actin staining images of MDCK cells on PIC/M hydrogels treated with cytochalasin D (f) and blebbistatin (g) after cell seeding for 96 h. Inhibitors were added on 48 h respectively. All images are representative of *n* = 3 independent biological experiments. Scale bar: 20 µm.

**Figure:**
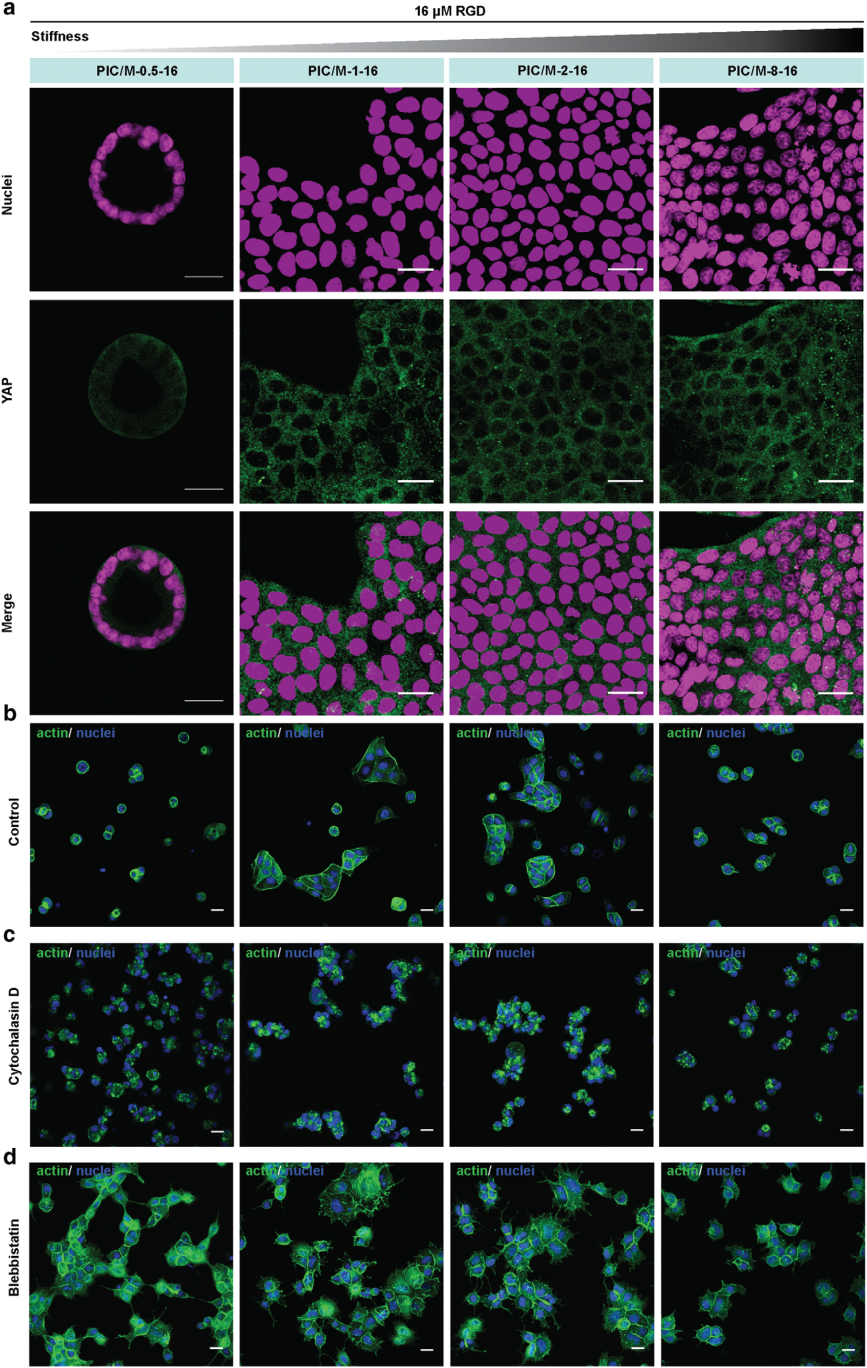

**Figure:**
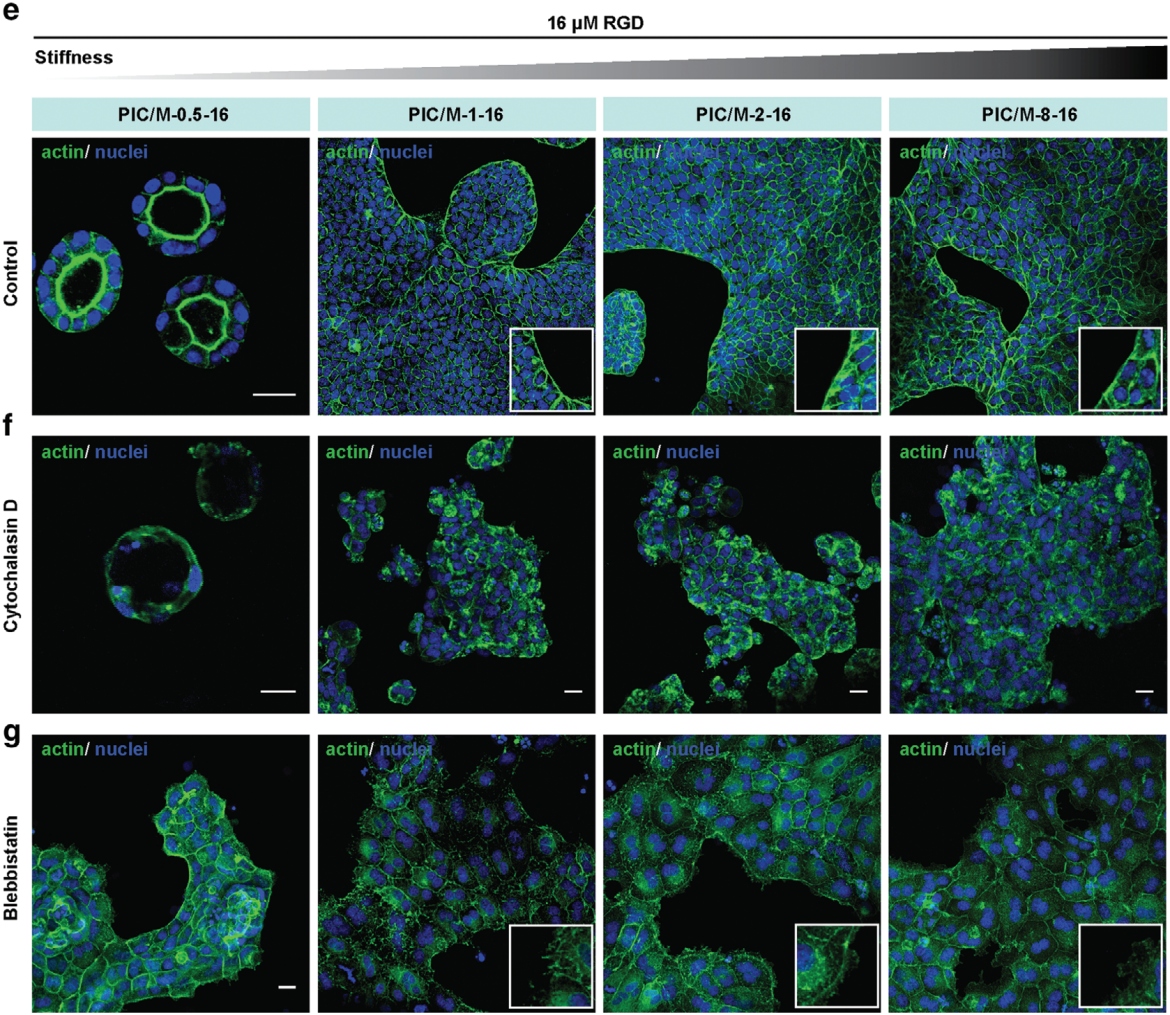

Actin contraction by the non‐muscle molecular motor myosin II generates the forces that drive morphogenesis into 3D cysts or monolayer sheets.^[^ ^51^, ^52^ ^]^ To further explore the role of the PIC/M hydrogel‐dependent F‐actin organization in MDCK morphogenesis, we studied the effects of inhibitors of non‐muscle myosin II (blebbistatin)^[^ ^53^ ^]^ and actin polymerization (cytochalasin D).^[^ ^54^ ^]^ In one experiment, we studied the formation of cellular structures by addition of the inhibitors during cell seeding, followed by fixation and analysis after 5 h (Figure 4b–d). In a second experiment, we focused on the maintenance of cellular structures by adding the inhibitors after morphogenesis has commenced (48 h after seeding) and analyzed after another 48 h (Figure 4e–g).

Early non‐muscle myosin II or actin polymerization inhibition of MDCK cells cultured on a soft substrate (PIC/M‐0.5‐16) prevented cyst formation altogether. Actin polymerization inhibition using cytochalasin D gave only cell clusters, and non‐muscle myosin II inhibition in blebbistatin‐treated cells led to clusters of cells exhibiting protrusions. Interestingly, the cells treated early with inhibitors on the stiffer substrates showed nearly identical behavior as those seeded on the soft substrates (Figure 4c,d), which implies that non‐muscle myosin II and actin may be involved in the mechanotransduction mechanism that is responsible for the morphological shift. From our results, we conclude that non‐muscle myosin II and actin jointly generate contractile forces necessary for 3D cyst formation.

Treatment of already formed cysts (on PIC/M‐0.5‐16) with cytochalasin‐D yielded deformed and poorly organized cysts compared to the non‐treated control (Figure 4f). After treatment with blebbistatin, cysts were no longer observed in the sample, and rather monolayers were found (Figure 4g). Similarly, on the stiffer substrates, MDCK cells treated with cytochalasin‐D or blebbistatin displayed uncontrolled and limited spreading, and interrupted actin localization (Figure 4f,g). The 2D monolayer morphology was maintained, but cells at the boundaries of the monolayer sheets showed many small protrusions (insets in Figure 4g), rather than the characteristic actin‐myosin cables (insets in Figure 4e). Taken together, the forces generated by actin and non‐muscle myosin II are important for cyst maintenance, creating smooth boundaries and producing coordinated movements in epithelial monolayers, which are important in tissue morphogenesis.^[^ ^6^, ^51^ ^]^

**Figure 5:**
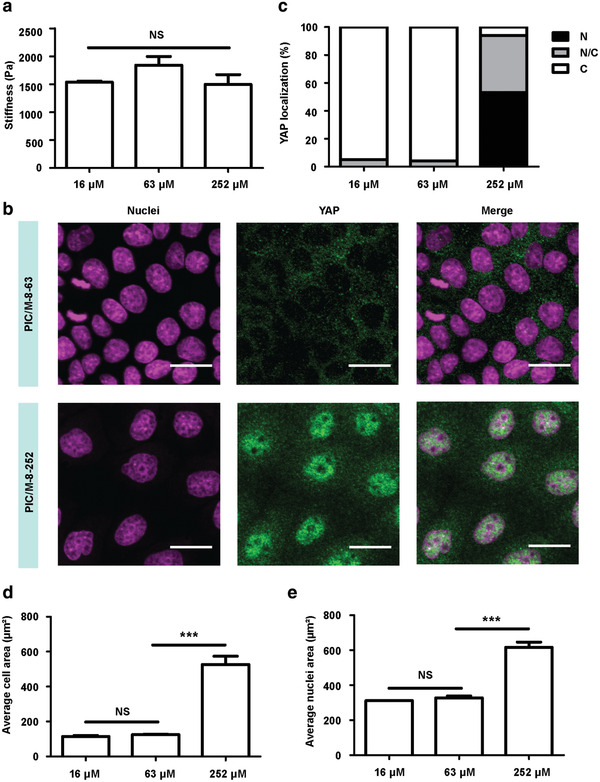
YAP translocation from cytoplasm to nuclei. a) Bulk stiffness characterization of PIC/M‐8 gels of different RGD density. The given values of *G′* are the averages of at least three independent measurements. b) Representative immunofluorescence staining images of YAP of MDCK cells on PIC/M‐8 substrates of different RGD density. All images are representative of *n* = 3 independent biological experiments. Scale bar: 20 µm. c) Fraction of cells on PIC/M hydrogels displaying preferential nuclear YAP localization (N, black), even distribution of YAP in nucleus and cytoplasm (N/C, gray), or cytoplasmic YAP (C, white). The YAP translocation results are based on scoring ≥2000 cells for each sample. d,e) The average quantitation of cell (d) and nuclei (e) area of MDCK cells on PIC/M‐8 substrate of different RGD density. The area quantification results are based on scoring ≥200 cells for each sample. NS = no significant, ***p < 0.001, one‐way ANOVA followed by Tukey's multiple comparisons test. Bars represent mean ± SEM.

It is striking that all mechanical effects observed so far, were not accompanied by nuclear translocation of YAP, even on a substrate with a storage modulus *G*′ as high as 1.5 kPa (PIC/M‐8‐16). We hypothesized that the low concentration of adhesive peptide only allows for minimal cell‐matrix interactions, which attenuates mechanical transduction. We then took full advantage of the synthetic matrix, where we can readily manipulate molecular parameters and we prepared hybrid matrices of PIC polymers with increased RGD concentrations: PIC/M‐8‐16, PIC/M‐8‐63 and PIC/M‐8‐252.^[^ ^55^ ^]^ The matrices with these RGD densities displayed a similar stiffness (**Figure** **5**a), but the cells on the gel with the highest RGD density (252 µm) displayed distinctly different behavior. For these cells, the majority of YAP translocated to the nucleus (Figure 5b,c).^[^ ^56^ ^]^ In addition, the average area of the cell and the nucleus increased significantly (Figure 5d,e; Figure S13, Supporting Information), most likely due to flattening of the cells as a response to the increased RGD mediated cell‐substrate interactions.^[^ ^18^, ^57^ ^]^

### 2.5 MDCK Morphogenesis in 3D Hybrid Cultures

**Figure 6:**
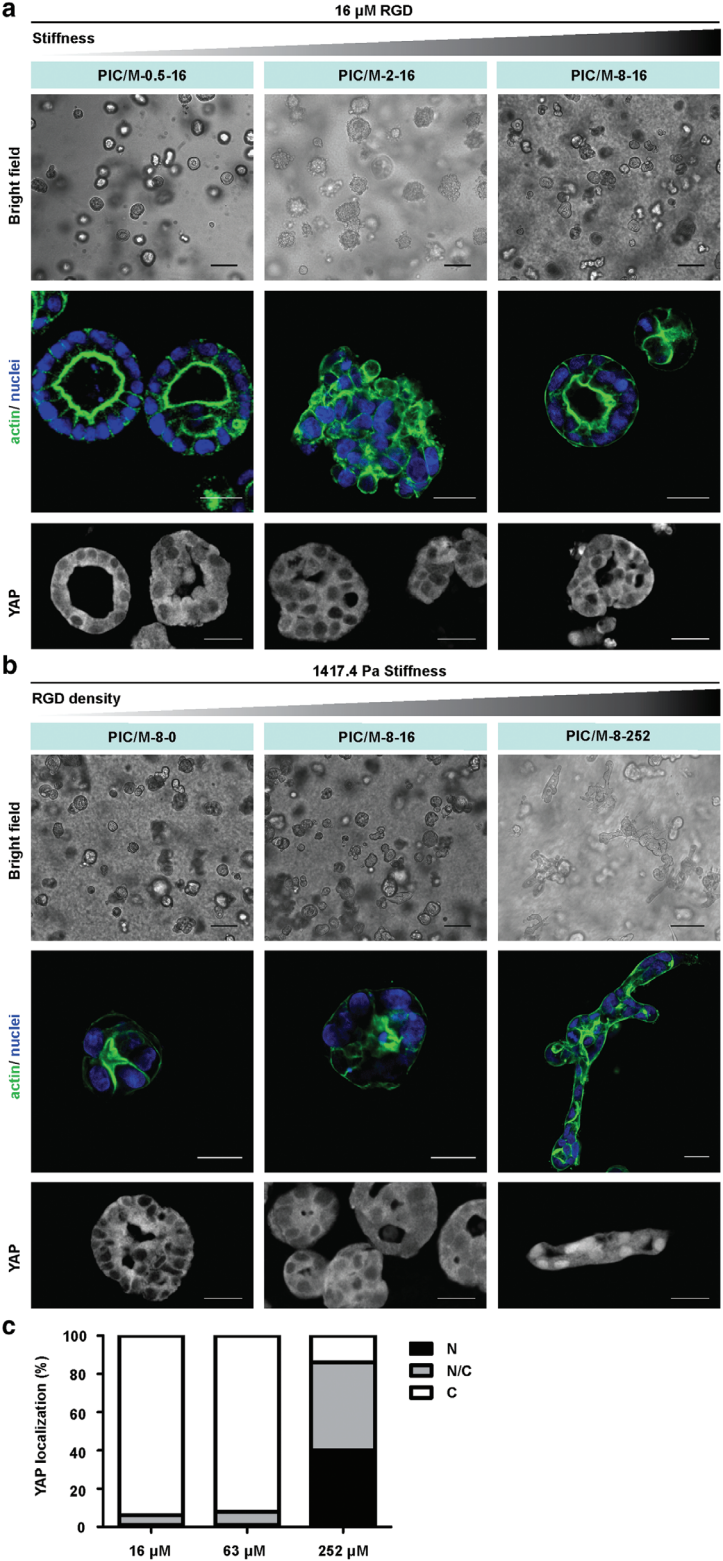
MDCK cell morphologies in 3D PIC/M hybrid matrices. a) Representative bright field (panel on the top), actin staining (panel in the middle) and YAP immunofluorescence staining (panel on the bottom) images of MDCK cells in PIC/M‐0.5‐16, PIC/M‐2‐16, and PIC/M‐8‐16. b) Representative bright field (panel on the top), actin staining (panel in the middle) and YAP immunofluorescence staining (panel on the bottom) images of MDCK cells in PIC/M‐8 of different RGD density. c) Proportion of cells on PIC/M‐8 of different RGD density displaying preferential nuclear YAP localization (N, black), even distribution of YAP in nucleus and cytoplasm (N/C, gray), or cytoplasmic YAP (C, white). The results are based on scoring ≥2000 cells for each sample. All images are representative of *n* = 3 independent biological experiments. Bright field scale bar: 50 µm. Staining scale bar: 20 µm.

Considering the reported differences in YAP activation in 2D and 3D matrices, we next studied MDCK morphogenesis and YAP localization in 3D hydrogels. MDCK cells (400 000 mL^−1^) were mixed with the cold matrix solution and subsequently gelled at 37 °C (PIC gels form within seconds after heating). Analogous to the 2D experiments, we independently varied the PIC concentration (**Figure** **6**a) and RGD density (Figure 6b). In the corresponding PIC only gels, we observe for all PIC and RGD concentrations the formation of well‐organized rounded cysts (Figure S14, Supporting Information).^[^ ^58^ ^]^ An overview of cell behavior in the 3D cultures is given in Table S2, Supporting Information.

After 4 days in the 3D culture, cells in PIC/M‐0.5‐16, PIC/M‐2‐16 and PIC/M‐8‐16, all formed round cell clusters (Figure 6a). Actin staining showed that in the softest matrix (PIC/M‐0.5‐16), these clusters were hollow, and were very similar to the cysts formed on the same matrix in the 2D experiment. The clusters in PIC/M‐2‐16 were solid and disorganized; those in the stiffest PIC/M‐8‐16 hollow again, at first sight similar to those in PIC/M‐0.5‐16. Note that in the corresponding 2D experiments on the stiffer PIC/M substrates, monolayers were formed. YAP staining results of the cells in the 3D cultures with low cell‐adhesive peptide density showed that the transcription factor is principally located in the cytoplasm (Figure 6a), analogous to the 2D experiments on the same gels.

We then kept the stiffness of the 3D matrix constant (*G′* ≈ 1500 Pa) and varied the RGD density between 0 and 252 µm, controlling the mechanical interactions between the cells and the (relatively stiff) matrix. For the cultures in PIC‐8‐16 and PIC‐8‐252, we observed the formation of round hollow cysts with the difference that for the cells in PIC‐8‐16 YAP resided in the cytoplasm and for those in PIC‐8‐252, YAP had translocated to the nucleus (Figure S15, Supporting Information). In the corresponding PIC/M hybrids, PIC/M‐8‐0 and PIC/M‐8‐16, also round cysts formed with YAP residing in the cytoplasm (Figure 6b). In PIC/M‐8‐252, at the highest RGD concentration (252 µm), tubular structures were formed (Figure 6b), which commonly requires the addition of specific biochemical cues, such as growth factors.^[^ ^46^ ^]^ The morphological change from spherical to tubular structures in the 3D cultures required Matrigel components, a stiff matrix and sufficient anchor sites to the matrix for mechanotransduction. Tubule formation coincided with YAP translocation and, likely, activation (Figure 6b). The results of our 2D and 3D experiments are fully consistent: YAP remains in the cytoplasm at low RGD levels, but translocates to the nucleus in PIC/M‐8‐252 (Figure 6c), indicative of activation of a YAP‐controlled mechanotransduction mechanism at these conditions.

Recently, other groups who studied epithelial cell behavior in and on hybrid hydrogels of Matrigel with either collagen^[^ ^18^ ^]^ or alginate^[^ ^16^ ^]^ presented seemingly contradictory results. In the collagen hybrids, an increase in the collagen concentration, aiming at increasing the mechanical properties but at the same time increasing cell the adhesion site density, resulted in YAP translocation to the nucleus.^[^ ^18^ ^]^ In contrast, increasing the alginate concentration in the hybrids, even to a storage modulus of 20 kPa, only resulted in cytoplasmic YAP.^[^ ^16^ ^]^ We argue that both results perfectly agree with our observations in the PIC‐Matrigel hybrid matrices: nuclear YAP in relatively stiff gels with sufficient cell adhesion sites and YAP that resides primarily in the cytoplasm for (stiff or soft) matrices with insufficient cell‐binding capacity. Despite the abundance of binding sites in the soft Matrigel component, it seems that a minimal RGD‐binding site density on the stiffer PIC/M component is crucial to initiate YAP activity.

## 3 Discussion

### 3.1 Controlling MDCK Morphogenesis in PIC‐Matrigel Hybrid Matrices

Our PIC/M matrices are a combination of a synthetic hydrogel with animal‐derived Matrigel. By tailoring the composition of the hybrids, we are able to conveniently and independently tune key parameters, such as stiffness and ligand densities. As Matrigel contains numerous binding sites for cell attachment, we were surprised to find that a limited RGD density (as low as 16 µm) on the PIC has a large effect on cell behavior. Apparently, the RGD on the PIC backbone is dominant in transferring the physical (mechanical) characteristics of the matrix.

Together, the results of our study show that both components in our PIC/M hydrogels play a crucial role to control cell behavior and morphogenesis. Integrins on the cell membrane are expected to interact simultaneously with the PIC scaffold and with Matrigel components, including laminin. The *α*_v_ *β*_6_ integrin, expressed by MDCK cells is the main RGD‐binding integrin and is directly involved in mechanotransduction and cell spreading.^[^ ^59^ ^]^ For polarized cyst formation, however, the *α*_v_ *β*_6_ integrin is dispensable and, rather, the *α*_3_ *β*_1_ integrin—one of the main laminin‐binding integrins—plays a key role.^[^ ^39^, ^40^ ^]^ We propose that in the hybrid gels, the PIC‐RGD network provides the necessary (tunable) mechanical cues, while Matrigel provides basement membrane components, in particular laminin, that after cellular remodeling drives lumen formation and apical‐basal polarization.^[^ ^39^, ^40^, ^41^ ^]^ Interestingly, *α*_v_ *β*_6_ integrin and the laminin‐binding *β*_1_ integrins are known to functionally cooperate in MDCK cells, as the *α*_v_ *β*_6_ integrin stabilizes *β*_1_ integrin‐containing FAs and recruit supporting mechanosensitive proteins.^[^ ^59^ ^]^ As such, initial binding of MDCK cells to the PIC‐RGD may potentiate the ability of the cells to interact with Matrigel components using the non RGD‐binding integrins. Those interactions likely also promote the assembly of laminin into a basement membrane, and consequently, contribute to polarized cyst morphogenesis. We note that we cannot exclude secondary effects from the formation of a hybrid hydrogel, such as the masking or capturing of bioactive factors of one of the components. These effects are intrinsic to hybrid matrices and are further aggravated by the rich and complex composition of Matrigel.

**Figure 7:**
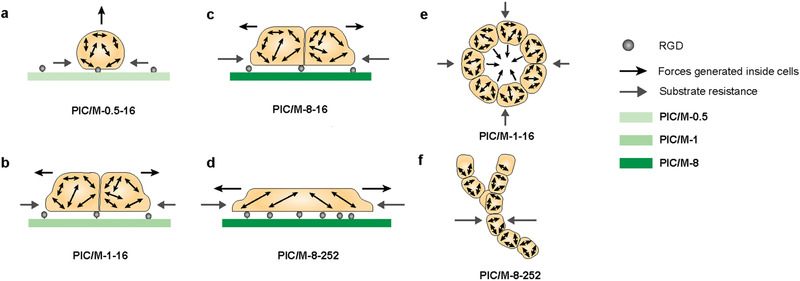
Schematic overview of morphogenesis drivers: forces generated inside MDCK cells and the substrate response to these cellular forces. 2D cell cultures: a) Soft matrices provide little interaction and intrinsic cellular forces give rise to cyst formation. b,c) On stiffer matrices, substrate interaction induces the formation of monolayers, but mechanotransduction is limited by the interaction density through integrin binding. d) On stiffer matrices with sufficient cell‐adhesive peptides, MDCK cells strongly adhere, form flattened monolayers and only then, the YAP mechanotransduction pathway is activated. 3D cell cultures: e) In softer matrices, limited cell‐ECM interactions and dominating intrinsic cellular forces result in cyst formation. f) In stiffer matrices with sufficient cell‐adhesive peptides, MDCK cells form tubular structures and with simultaneous YAP translocation.

Previous research has suggested that for modeling tissue morphogenesis in vitro, cells should be cultured in an environment that permits free growth and remodeling of their environment.^[^ ^60^ ^]^ Gehler et al. showed that breast epithelial cells undergo ductal morphogenesis when cultured in a soft low‐density matrix but not when the same matrix is crosslinked and under higher tension.^[^ ^61^ ^]^ The authors proposed that a stiffer ECM clusters the integrins more effectively, which activates proliferation and cell contractility to disrupt normal morphogenesis of breast epithelia. Our results indicate that MDCK cells seeded on soft gels (PIC/M‐0.5‐16, **Figure** **7**a) experience limited (mechanical) interaction with the substrate, but display sufficient contractile forces for the formation and maintenance of 3D cysts, analogous to what is commonly observed in Matrigel.^[^ ^45^ ^]^ One can draw a parallel with the early stages of embryonic morphogenesis where intercellular interactions dominate over matrix effects.^[^ ^62^ ^]^ Inhibiting force generation or transmission straightforwardly prevents desirable morphogenesis. With a stronger mechanical response from the substrate (Figure 7b–c), a morphological shift is observed. Stiffer substrate provides the initial trigger for a cascade of RGD based mechanotransduction processes that influence cell spreading, FAs formation and cell‐fate decisions. The actomyosin contractile forces are (also) directed towards the substrate, resulting in well‐defined monolayers. For these substrates, the response to cytoskeletal contraction depends on the balance between the contractile forces and the substrate stiffness, or more properly, the efficiency of force transduction determined by the substrate stiffness and the concentration cell‐adhesive peptides. When the substrate stiffness is increased and the mechanical sensing is efficient through a sufficiently high density of cell‐matrix interactions (Figure 7d), a change in cell morphology can be observed when in the monolayers, the cells and nuclei flatten.

Our results also provide information on mechanotransduction pathways involved in MDCK morphogenesis at physiological stiffness levels. Only at “high” stiffness (1.5 kPa) and “high” RGD concentrations, the YAP pathway is activated as primarily nuclear YAP is then observed. At lower stiffness or RGD availability, YAP is cytoplasmic and the differences in morphology induced by differences in the substrate properties originate from a YAP‐independent mechanotransduction pathway. Although our studies show that actin and non‐muscle myosin II are involved, the details of this parallel signaling pathway remain unclear.

The mechanotransduction analysis in 3D cultures give results that are analogous to what is seen in 2D (Figure 7e,f): at low PIC and RGD concentrations, YAP is localized in the cytoplasm and only at the highest concentrations of both, nuclear YAP is observed. In 3D, this shift is concurrent with a morphological shift; the formation of tubular structures. Cells in pure PIC‐8‐252 hydrogels and PIC/M‐8‐252 both display YAP translocation from cytoplasm to nuclei (Figure S15, Supporting Information), but only the latter forms tubular structures, once again indicated that the presence of Matrigel facilitates matrix remodeling and morphogenesis.

## 4 Conclusion

Morphogenesis is crucial in tissue formation and controlling the underlying force‐driven processes through well‐defined substrates and 3D matrices is of great value. Here we present a novel cell culture system based on PIC‐Matrigel hybrid matrices composed of a natural matrix and synthetic hydrogels that is able to direct MDCK morphogenesis and can be used to explore the elements that are involved in mechanical transduction. In our hybrids, MDCK cells develop into a broad range of morphologies, ranging from distinct round hollow cysts to extended 2D monolayers with the correct polarization, and even—in 3D—into tubular structures. By manipulating the PIC characteristics, the matrix stiffness and density of RGD based cell‐binding ligands can be tailored independently, while keeping the concentrations of other (soluble) factors from Matrigel constant. Our results confirm that matrix stiffness is a key factor, but they also highlight the important role of appropriately placed cell‐binding sites in or on the network. We find that at least two mechanotransduction mechanisms are active. In one, which determines the morphological shift from 3D cyst to monolayer, YAP is not translocated to the nucleus. In the second mechanism, YAP clearly is involved, which results in strong anchoring to the substrate that leads to a flattened morphology. We find analogous results in 2D cultures and in 3D experiments, where the gels more closely mimic the in‐vivo 3D microenvironment. Interestingly, we find the formation of tubular morphologies after YAP activation in our 3D culture, which commonly requires additional factors. In short, our work illustrates how external physical factors can be used to regulate morphogenesis and how beneficial it is to study these effects in well‐defined matrices.

## 5 Experimental Section

##### Synthesis of and Preparation of PIC‐Azide, PIC‐RGD and Cy3‐Functionalized PIC

The synthetic hydrogels are based on tri(ethylene glycol)‐grafted polyisocyanides.^[^ ^19^ ^]^ The syntheses of azide‐functionalized PICs and RGD‐functionalized PICs, summarized in Figure S1, Supporting Information, follow previously reported procedures.^[^ ^28^ ^]^ The co‐polymerization of tri(ethylene glycol)‐grafted isocyano‐(*D*)‐alanyl‐(*L*)‐alanine monomer **1** and the azide‐appended monomer **2** (molar ratio of **1**:**2** = 99:1) was catalyzed by nickel perchlorate (total monomer: catalyst ratio for all polymers was 2000:1) and continued until FT‐IR spectroscopy indicated that all free isocyanide monomers were all consumed (24 h, Figure S16a, Supporting Information). After purification, the polymer was obtained as a light yellow solid, which was then sterilized (UV, 5 min treatment) and dissolved in the desired concentration in sterile HBSS (Sigma Aldrich, Cat. #H4891, prepared by dissolving Hank's Balanced Salts (1 g in 1 l sterile H_2_O). After soaking at 4 °C overnight, the mixture was shaken vigorously for a few seconds. If air bubbles were present, these were removed by centrifuging at 4 °C, 1500 rpm 30 s, after which a transparent solution was formed. When the temperature is raised above the gelation point, the cold transparent solution immediately forms a soft, elastic gel. For the entire study, one large batch of PIC solution in HBSS was prepared, which was then aliquoted and frozen at −20 °C until use. Before each experiment, the frozen gels were first placed on ice to thaw.

##### Synthesis of and Preparation of PIC‐Azide, PIC‐RGD and Cy3‐Functionalized PIC‐RGD‐Functionalized PIC Gels

The azide groups on the polymers were reacted with acetylene‐equipped cell‐binding peptides (RGD) through the highly efficient^[^ ^63^ ^]^ strain‐promoted azide‐alkyne cycloaddition reaction following literature procedures.^[^ ^28^, ^43^ ^]^ Note that the concentration of the RGD peptide is controlled by mixing the RGD‐functionalized polymer with its precursor, the azide‐appended polymer. Bundle formation in the gel phase ensures a good distribution of RGD peptides over the network.

##### Synthesis of and Preparation of PIC‐Azide, PIC‐RGD and Cy3‐Functionalized PIC‐Cy3‐Functionalized PIC Gels

DBCO‐Cy3 (0.84 µL, 20 mm in DMSO, Sigma Aldrich, # 777366) was mixed thoroughly with a cold azide‐functionalized PIC solution (1 mL, 16 mg mL^−1^ in HBSS) and left for 30 min on ice to allow full conjugation.

##### PIC/M Hybrid Hydrogel Formation and Characterization

Matrigel (cat. #354234; Corning) was purchased to construct the hybrid hydrogels and used at a final concentration of 4.4 mg mL^−1^ for all experiments. For the PIC/M hydrogels, the desired amounts of cold stock solutions (16 mg mL^−1^) of PIC with and without RGD mixed with cooled Matrigel and additional HBSS was added when necessary. After stirring well, at low temperatures, the PIC/M solutions are ready for use.

##### Rheology and Viscometry Measurements

Rheological measurements were performed on a stress‐controlled rheometer (Discovery HR‐2, TA Instruments) using a steel parallel plate geometry with a diameter of 40 mm and a gap of 500 µm. Cold gel solutions were loaded on the rheometer plate, which was set to 5 °C. The shear or storage modulus *G′* was measured in oscillatory deformation (amplitude *γ* = 0.04, a frequency *ω* = 1.0 Hz) in a temperature ramp (5 to 37°C with 1 °C min^−1^). The given values *G′* were recorded at 37 °C and are the averages (and Standard error of the Mean, SEM) of at least three independent measurements. Representative rheology heating curves of the PIC/M hybrid hydrogels are shown in Figure S17, Supporting Information; a full overview of storage moduli is given Figure S3, Supporting Information. Note that RGD conjugation does not considerably change the mechanical properties of the PIC gels or the hybrids. The nonlinear regime was studied by applying a constant pre‐stress (*σ*_0_ = 0.2–500 Pa) to the samples and measure the mechanical properties using a small superposed oscillatory stress (*ω* = 10–0.1 Hz) according to the pre‐stress protocol described before.^[^ ^20^ ^]^ Data are given in Figures S4 and S5, Supporting Information. The viscosity average molecular weight *M*_v_ of the azide‐appended polymer was measured by viscometry (Figure S16b, Supporting Information) in acetonitrile, yielding *M*_v_ = 419 kDa, from which the polymer length (*L*) was calculated.^[^ ^10^ ^]^

##### Immunofluorescence Staining of Matrigel

To prepare the PIC/M hydrogels, Cy3‐functionalized PIC and Matrigel were homogeneously mixed in the desired ratio. A drop of PIC/M solution (50 µL) was cast on the bottom of a chamber slide and left at 37 °C in the incubator for 1 h to allow for full gelation. They were then blocked with 2% bovine serum albumin (BSA) in PBS for 1 h at room temperature. After that, the samples were incubated in primary antibodies against laminin (Sigma Aldrich, #L9393, 1:10) in 1% BSA solution for 2 h at room temperature. They were washed with PBS, and subsequently incubated with Alexa 488‐conjugated secondary antibodies (Invitrogen, Cat. #A21206, 1:400) for 1 h at room temperature. Then, the samples were mounted in PBS, and images were acquired using an Olympus FV1000 confocal microscope. The acquired images were processed by Fiji.

##### Cell culture

Madin‐Darby Canine Kidney (MDCK) cells were cultured in MEM (GIBCO, Life Technologies) with 10% FBS, 1% L‐glutamine and antibiotics.

##### Cell culture—2D Cell Culture

According to previous descriptions, PIC/M hydrogels were obtained by mixing the appropriate PIC gels, Matrigel and HBSS in the desired ratio. After mixing, the gels (100 µL) were placed in an 8‐well chamber slide, and left in the incubator for 1 h to allow gel formation. In the meantime, the cells were trypsinized, counted and resuspended in fresh growth medium. Then, 700 µL of cell suspension with a density of 20 000 cells/well were seeded on top of the gelled PIC/M.

##### Cell culture—3D Cell Culture

PIC/M hydrogels of different parameters were prepared according to previous description. Then, cells were trypsinized, counted, resuspended in limited HBSS, and 1 volume of cell HBSS suspension was mixed with 50 volumes of the ECM mix to obtain 20 000 cells in 50 µL. After thorough mixing, the gels with the cells were seeded in drops in a 24 well plate. After 1 h gelation in the incubator, the wells were supplemented with normal growth medium.

##### Inhibitor Treatment

The pharmacological agents used were 50 µm blebbistatin (Merck, # 203390–5MG) and 1 µm cytochalasin D (Sigma Aldrich, #C2618). The MDCK cells were exposed to each pharmacological agent at 0 h (for 5 h until analysis) or 48 h (for 48 h until analysis) after seeding the cells on top of PIC/M hydrogels.

##### Immunofluorescence Analysis

For 3D cultured cell clusters immunofluorescence analysis, MDCK cell clusters generated within the PIC/M hydrogels were fixed with 4% paraformaldehyde (PFA) in PBS at room temperature for 1 h. The resulting pellet was incubated in 1.5% eosin at room temperature for 5 min. After a quick wash with PBS, the pellet was resuspended in a 2.25% agar solution at 80–90 °C. The hot agar solution with the cells was centrifuged (7200 rpm, 2 min) to allow the pellets to settle at the bottom. The solidified agar solution with cells was embedded in paraffin. Then, 2 µm thick sections were cut using a microtome, mounted onto superfrost slides and dried overnight at 37 °C. After deparaffinization with Histochoice (VWR, Cat. #H103‐4L, twice for 10 min each), the samples were rehydrated with 100% (2 × 1 min), 96% (2 × 1 min), and 70% (1 × 1 min) ethanol, followed by washes with tap water (2 × 1 min). Samples were then heated for 15 min in citrate buffer (pH 6.0, Dako, Cat. #S1699) in a microwave oven for antigen retrieval. After cooling to room temperature for 1 h, the samples were blocked in 2% BSA in PBS at room temperature for 1 h. Primary antibody incubation against rabbit YAP (CST, Cat. #14074, 1:200) was performed in 1% BSA in PBS at room temperature for 2 h. Alexa 488‐conjugated secondary antibodies (Invitrogen, Cat. #A21206, 1:400) in 1% BSA/PBS were incubated at room temperature for 1 h, followed by three PBS washes. Immunofluorescence experiments were performed with negative controls where the relevant isotype was added (rabbit isotype: Cell Signaling Technology, Cat. #3900, 1:15000). The samples were then incubated with DAPI (5 µg mL^−1^) at room temperature for 10 min, followed by three PBS washes. The slides were mounted in anti‐fade medium (Fluoromount W for microscopy, Serva), and images were acquired using a Leica DM6000 microscope (Leica). Alexa 488‐conjugated phalloidin (Life Technologies) was used at a dilution of 1:100 in 1% BSA to visualize F‐actin microfilaments and images were acquired using an Olympus FV1000 confocal microscope. Acquired images were processed by Fiji.

For 2D cultured MDCK cells immunofluorescence analysis, cysts generated on PIC/M‐0.5 were fixed with 2% PFA with 0.2% glutaraldehyde (to minimize or prevent depolymerization of Matrigel) for 10 min, following by further washes with PBS. The cells were permeabilized with 0.2% triton X‐100 in PBS for 1 h, followed with another 1 h of blocking with 2% BSA in PBS. The primary antibody incubation was performed in 1% BSA in PBS at room temperature for 2 h. The following primary antibodies were used: rabbit YAP (CST, Cat. #14074, 1:200), mouse gp 135 (a gift from Dr. Mirjam Zegers, 1:500), rabbit E‐cadherin (CST, Cat. #3195, 1:200) and mouse paxillin (a gift from Dr. Mirjam Zegers, 1:200). The secondary antibody incubation was in 1% BSA in PBS at room temperature for 1 h, followed by three PBS washes. Alexa 488‐ (Invitrogen, Cat. #A21206, 1:400) or 568‐ (Invitrogen, Cat. #A11031, 1:400) conjugated secondary antibodies were used. All immunofluorescence experiments were performed with negative controls where the relevant isotype was added (Mouse isotype: Biolegend, Cat. #400102, 1:500; rabbit isotype: Cell Signaling Technology, Cat. #3900, 1:15000). The samples were then incubated with DAPI (5 µg mL^−1^) at room temperature for 10 min, followed by three PBS washes. The samples were mounted in PBS. Alexa‐488 conjugated phalloidin (Life Technologies) was used 1:100 in 1% BSA to visualize F‐actin microfilaments. Images were acquired using an Olympus FV1000 confocal microscope. Acquired images were processed by Fiji.

##### YAP Localization Quantification—2D culture

YAP localization of PIC/M‐0.5/1/2/8‐16 was easy to quantify as YAP in almost all of cells is in the cytoplasm. Cells on the edge of monolayer sheets were difficult to distinguish and were scored as evenly distributed in nucleus and cytoplasm (N/C, gray). For cells that had YAP in the nuclei (in PIC/M‐8‐252), immunofluorescence images of YAP at 40× magnification from at least 5 randomly selected fields were taken. Then the YAP localization of each cell was scored manually. At least 3 independent gels are analyzed.

##### YAP Localization Quantification—3D culture

For each independent gel, immunofluorescence images at 40× from at least 5 randomly selected fields were taken. The distribution was quantified by scoring cells that display preferential nuclear (N, black), evenly distributed in nucleus and cytoplasm (N/C, gray), or cytoplasmic (C, white) YAP localization in each image. The given values are averages of at least 3 independent gels for each condition.

##### Nuclei Area Quantification

Immunofluorescence images of YAP (40× magnification) from at least 5 randomly selected fields were taken. Then, using Fiji, images were thresholded and filtered and the number of nuclei and their area were automatically quantified. The results are based on scoring ≥200 cells for each sample.

##### Cell Area Quantification

At least 5 randomly selected fields of YAP staining with confluent cells were acquired. Cell numbers were quantified based on the number of nuclei. Then the average cell area was calculated based on the area of the selected field and the number of nuclei. The results are based on scoring ≥200 cells for each sample.

##### Proliferation Assay

Culture medium was gently removed and new medium supplemented with cell proliferation reagent WST‐1 (Roche) at a final concentration 1:10 (WST‐1 stock solution: total working solution) was added and the culture plates were incubated at 37 °C, 5% CO_2_ for 2 h. The absorbance was measured at *λ* = 450 nm with a plate reader (Perkin Elmer 1420 Multilabel Counter). All samples were measured in triplicate.

##### Statistics

GraphPad Prism 8 and Cellprofiler were used for the statistical analysis of the data presented in this work. Presentation and pre‐processing: Data in images are reported as mean ± SEM. The statistical significance in Figures 1e,f and 5a,d,e; Figures S8, S11a, and S12, Supporting Information, was determined using one‐way ANOVA followed by Tukey's multiple comparisons test, NS = not significant, *0.01 < P< 0.05, **P < 0.01, ***P < 0.001. The statistical significance in Figure S3, Supporting Information, was determined using unpaired two‐sided *t*‐test, NS = not significant. The data in Figure S8, Supporting Information was normalized to absorbance of WST‐1 without cells. Sample sizes are given in the captions of the figures.

## Conflict of Interest

The authors declare no conflict of interest.

## Author Contributions

P.N.S. and P.H.J.K. contributed equally to this work. Y.Z. performed the experiments. A.N., A.E.R., P.N.S., M.M.P.Z., and P.H.J.K. supervised the project. Y.Z., P.N.S., M.M.P.Z., and P.H.J.K. wrote the manuscript. All authors provided critical feedback and helped shape the research, analysis, and manuscript.

## Data Availability

All data supporting the results of this study are available in the article and Supporting Information, or from the corresponding author on reasonable request.

## Supporting information

Supporting Information

Supporting Information

Supporting Information

Supporting Information

Supporting Information

Supporting Information
