# Supplementary material for: Tunable Hybrid Matrices Drive Epithelial Morphogenesis and YAP Translocation
Source: Adv Sci (Weinh). 2020 Dec 11;8(2):2003380. doi: 10.1002/advs.202003380 (PMC7816720; doi:10.1002/advs.202003380)
Supplement: Supplementary file 1 — Supporting Information [file ADVS-8-2003380-s001.pdf]

© 2020 Wiley-VCH GmbH

ADVANCED SCIENCE
Open Access

Supporting Information

for *Adv. Sci.,* DOI: 10.1002/advs.202003380

Tunable hybrid matrices drive epithelial morphogenesis and YAP translocation

Ying Zhang, Mirjam M.P. Zegers, Anika Nagelkerke, Alan E. Rowan, Paul N. Span*, Paul H. J. Kouwer*

Supplementary information

Tunable hybrid matrices drive epithelial morphogenesis and YAP translocation

Ying Zhang, Mirjam M.P. Zegers, Anika Nagelkerke, Alan E. Rowan, Paul N. Span, Paul H. J. Kouwer**

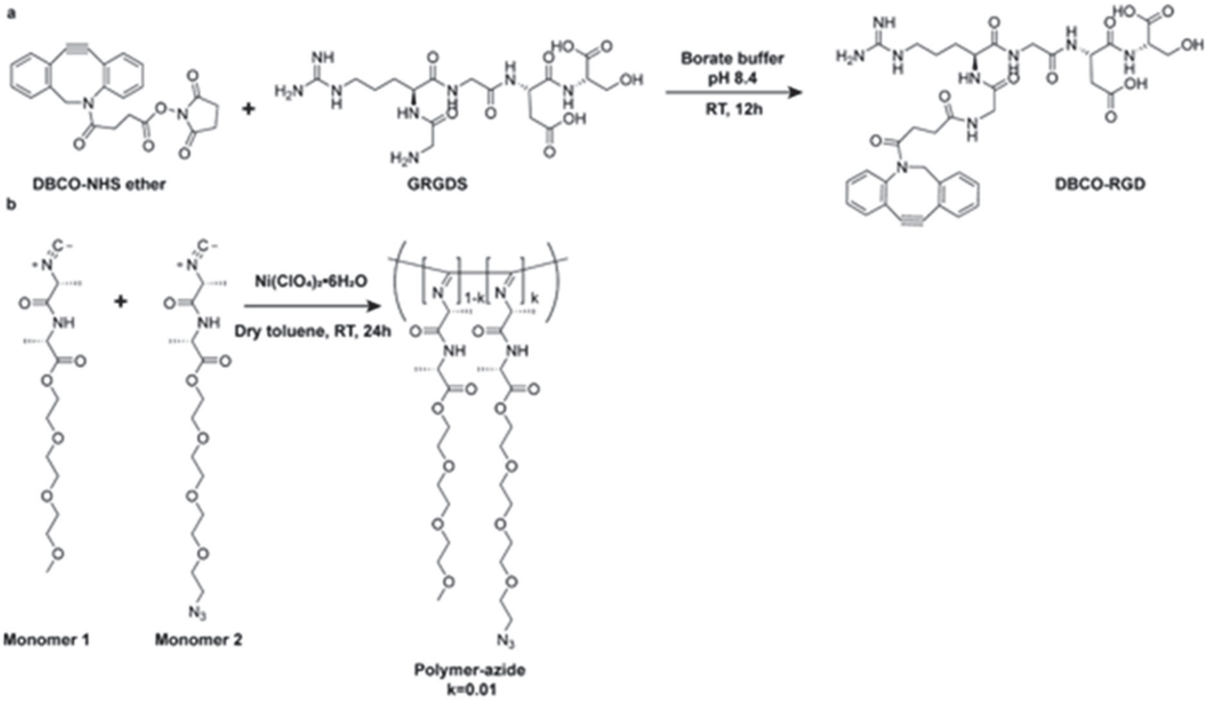

Figure S1. (a) Synthesis of intermediate DBCO-RGD; (b) Polymerization of PIC-azide. The distribution of N_3 groups is random with an average of every 14-18 nm along the polymer chain. RT = room temperature.

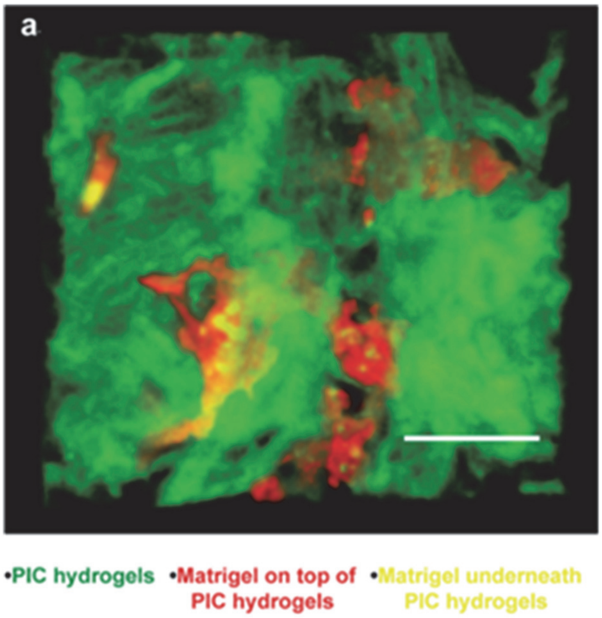

Figure S2. Representative confocal images displayed Matrigel in different positions indicating a tightly mixed morphology. Some of the Matrigel (green) is on top of PIC gels (red) or underneath of PIC gels. Scale bar: 50 µm. A clearer image is painted by confocal stack Movies 1 and 2.

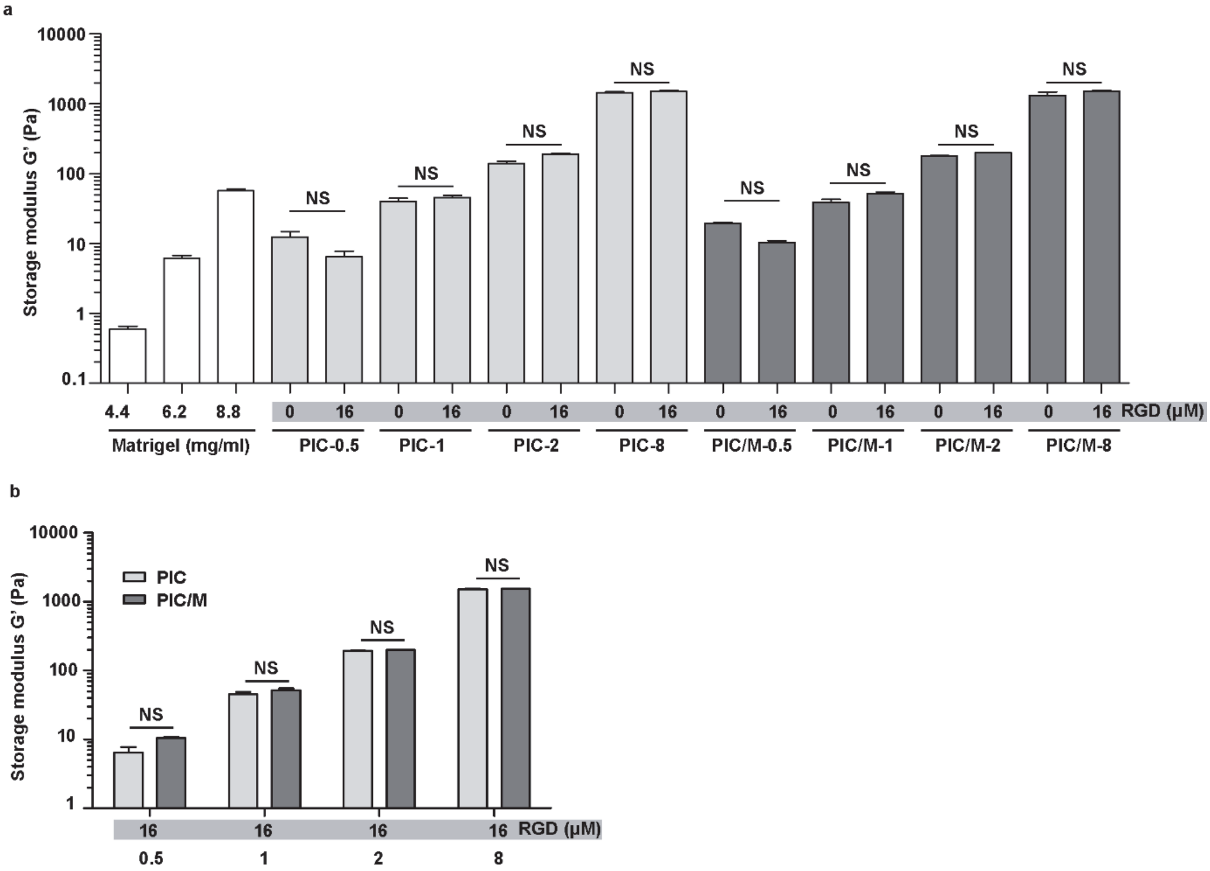

Figure S3. (a) Shear or storage moduli *G*′ of Matrigel, PIC gels and PIC/M hydrogels at different concentrations and ratios, indicating the influence of RGD conjugation. (b) Storage modulus *G*′ of PIC gels and PIC/M hydrogels

at different PIC concentrations, comparing the contribution of Matrigel. Values and error bars are the average of three independent measurements ± standard deviation. Statistics: unpaired two-sided t test, NS = not significant. Bars represent mean ± SEM.

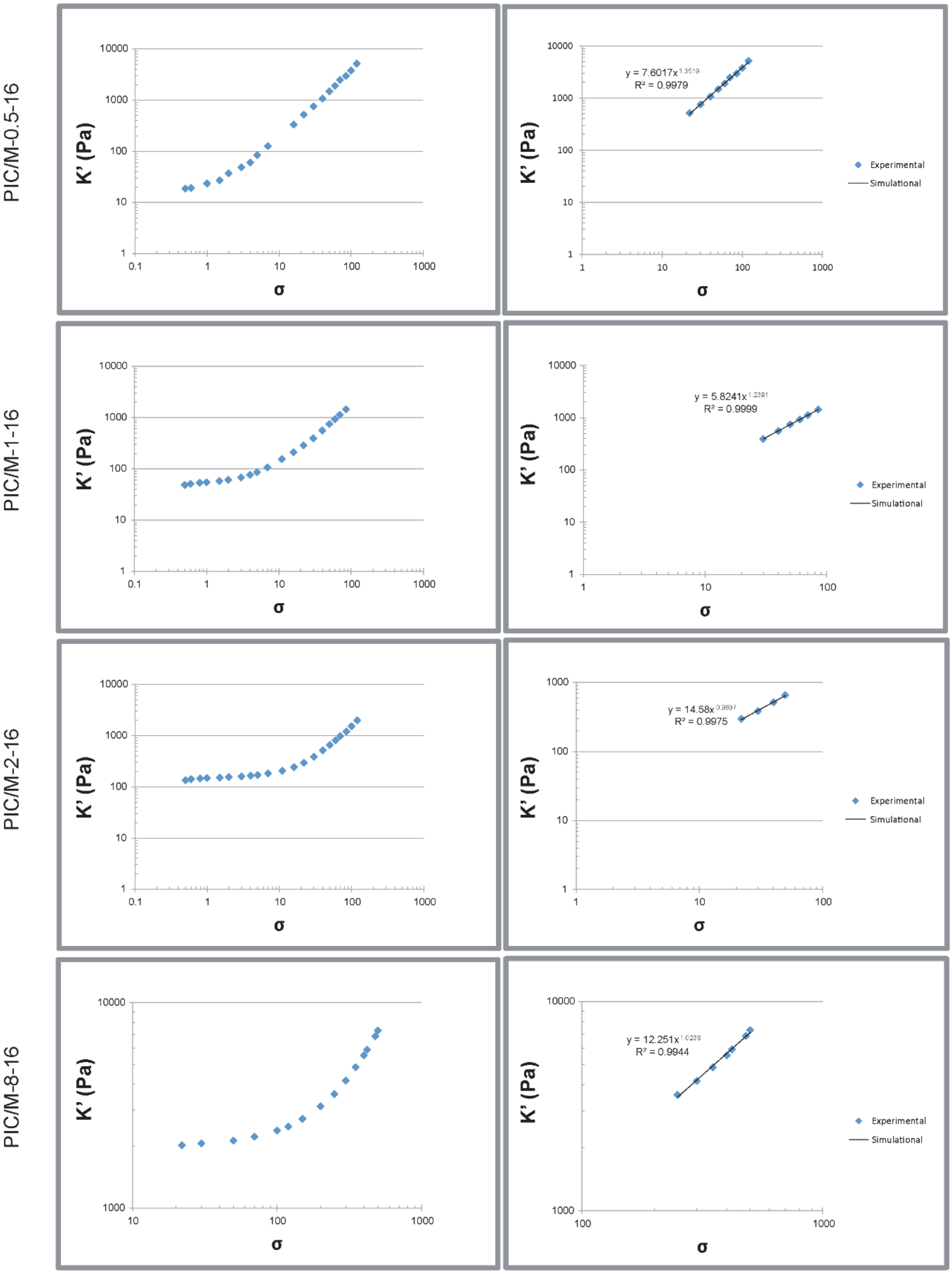

Figure S4. Representative nonlinear mechanical properties of PIC/M-0.5-16, PIC/M-1-16, PIC/M-2-16 and PIC/M-8-16, represented as the differential modulus *K'* as a function of applied pre-stress *σ*.

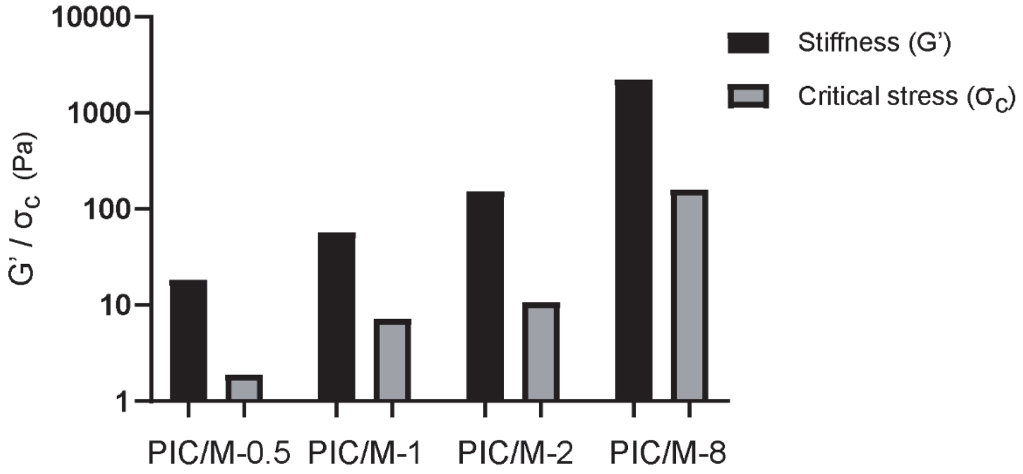

Figure S5. Storage modulus and critical stress (defined as the onset stress of stiffening) of PIC/M hydrogels. The biomimetic strain stiffening behavior of PIC gels is maintained in the PIC/M hydrogels.

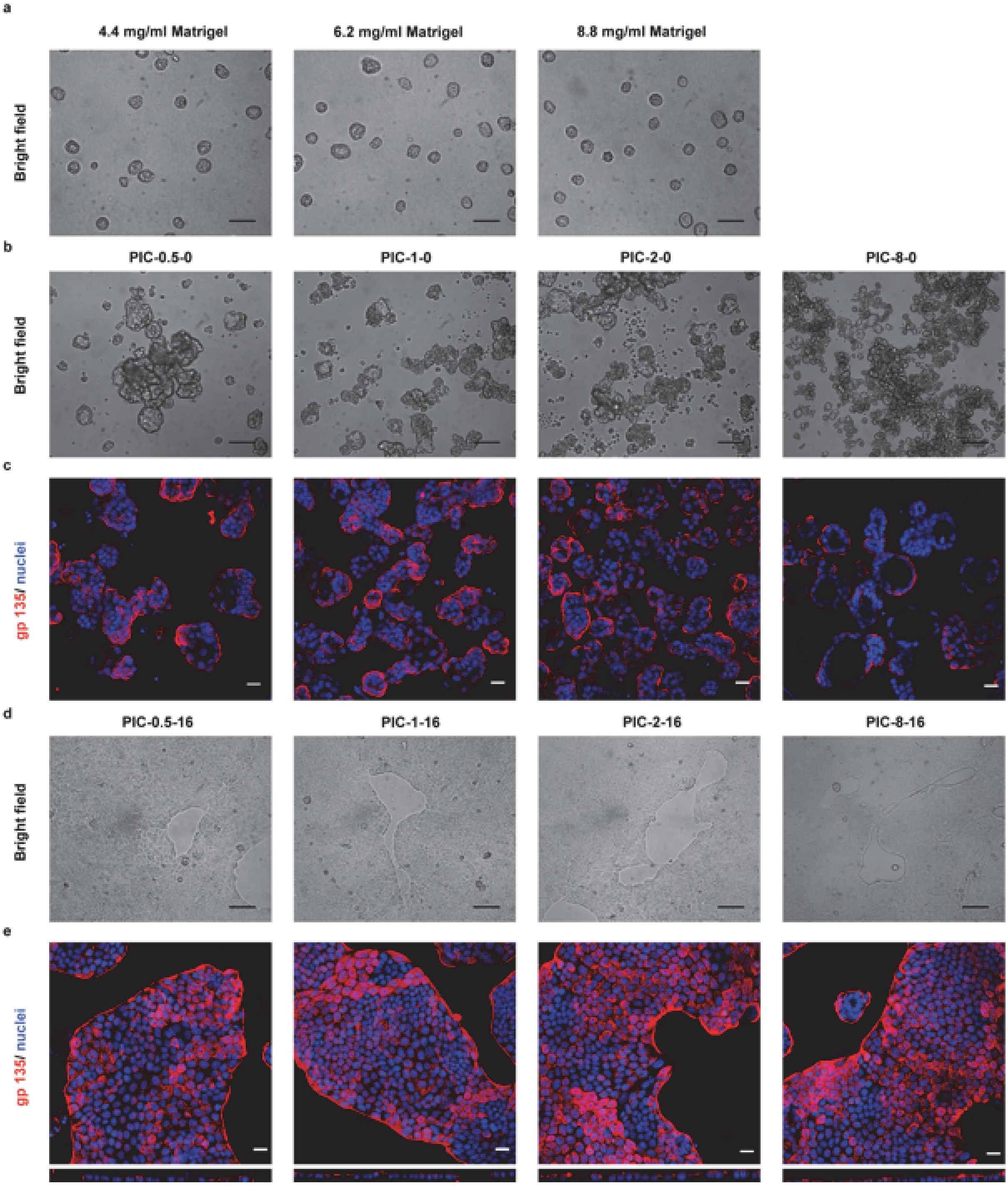

Figure S6. (a) Representative bright field images of MDCK cells on Matrigel (seeded with 2% of Matrigel in the medium). (b-c) Representative bright field images (b) and gp135 (red) immunofluorescence images (c) of MDCK cells on PICs without RGD conjugated. (d-e) Representative bright field (d) and gp135 (red) immunofluorescence images (e) of MDCK cells on PIC gels with 16 µM RGD conjugated. All images are representative of n = 3 independent biological experiments. Bright field scale bar: 100 µm. Fluorescence images scale bar: 20 µm.

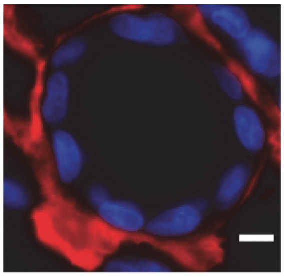

Figure S7. MDCK morphogenesis on PIC/M-0.5-16 showing laminin-111. Scale bar: 10 µm.

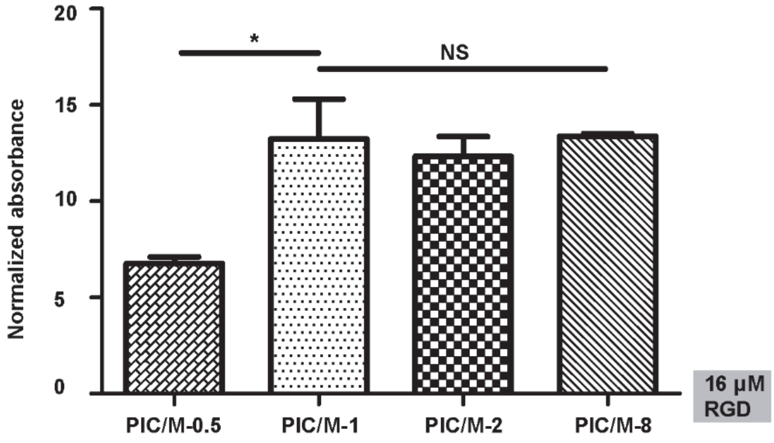

Figure S8. Proliferation assay of MDCK cells on different PIC/M hydrogels on day four after seeding, normalized to absorbance of WST-1 without cells.[1] Data obtained from n = 3 experiments. Statistics: one-way ANOVA followed by Tukey's multiple comparisons test. NS = not significant, * 0.01<P<0.05. Bars represent mean ± SEM.

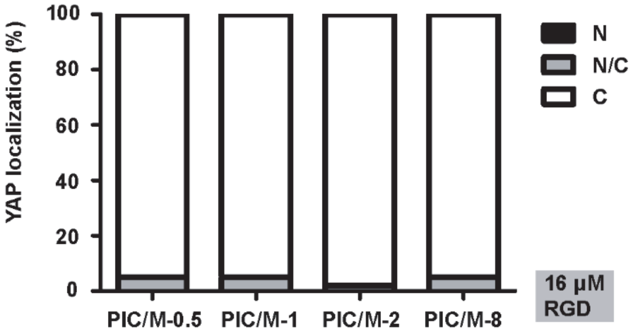

Figure S9. Proportion of cells on PIC/M hydrogels displaying preferential nuclear YAP localization (N, black), even distribution of YAP in nucleus and cytoplasm (N/C, gray), or cytoplasmic YAP (C, white). The results are based on scoring ≥2,000 cells for each sample.

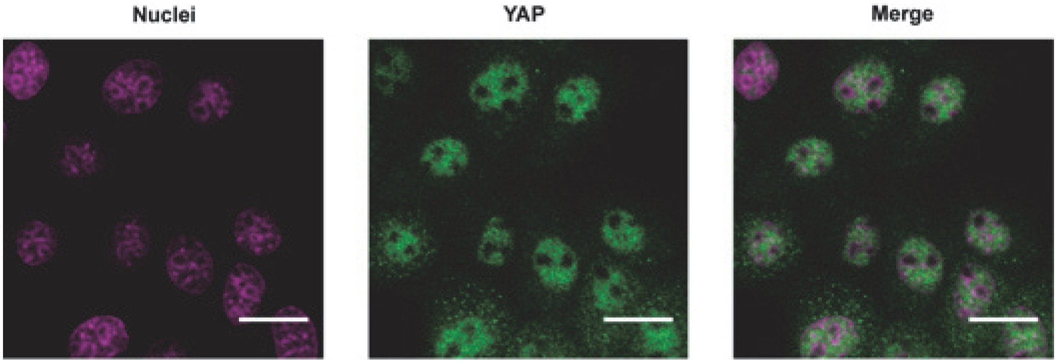

Figure S10. YAP translocation from cytoplasm to nuclei. Representative immunofluorescence images of YAP (green) and nuclei (magenta) of MDCK cells on PIC/M-8-252. All images are representative of at least n = 3 independent biological experiments. Scale bar: 20 µm.

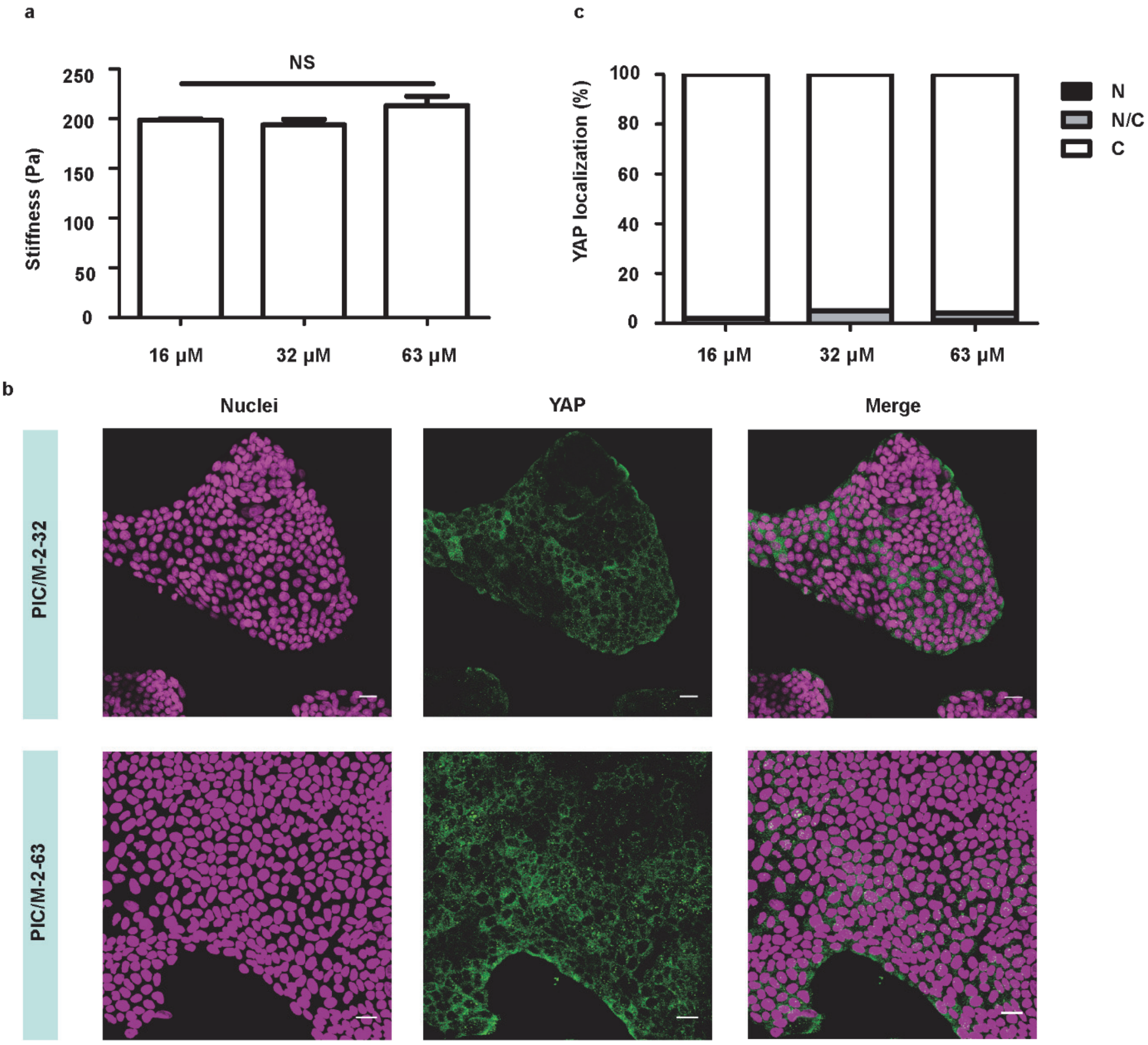

Figure S11. YAP remains in cytoplasm in PIC/M-2. (a) Bulk stiffness characterization of PIC/M-2 gels of different RGD density. The given values of *G'* are the averages three independent measurements; the bars represent SEM. Statistics: one-way ANOVA (followed by Tukey's multiple comparisons test). NS = not significant. (b) Representative immunofluorescence staining images of YAP of MDCK cells on PIC/M-2 substrates of different RGD density. All images are representative of n = 3 independent biological experiments. Scale bar: 20 µm. Scale bar: 20 µm. (c) Proportion of cells on PIC/M hydrogels displaying preferential nuclear YAP localization (N, black), even distribution of YAP in nucleus and cytoplasm (N/C, gray), or cytoplasmic YAP (C, white). The results are based on scoring ≥2,000 cells for each sample. Bars represent mean ± SEM.

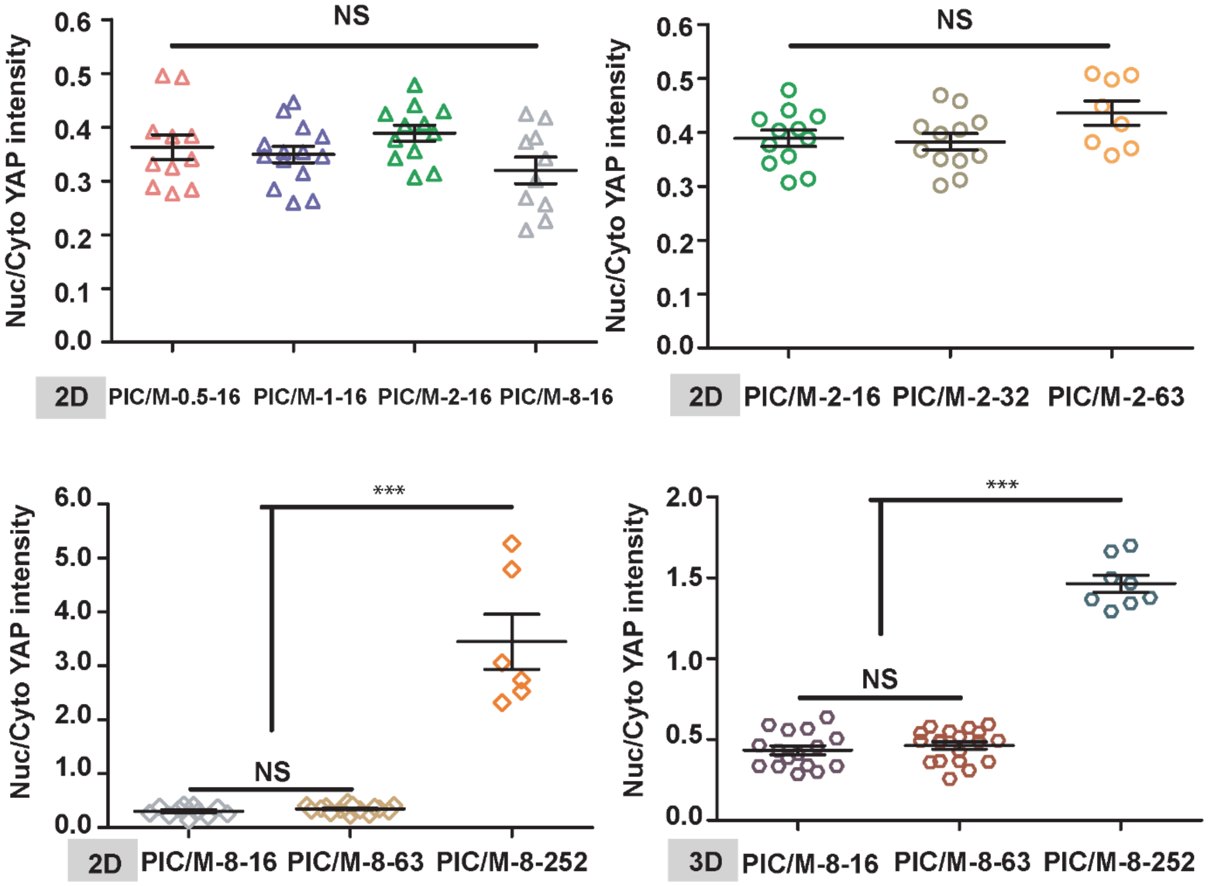

Figure S12. YAP quantification from 2D and 3D cultures. Cell Profiler was used to quantify the intensity ratio of nuclear and acytoplasmic YAP from immunofluorescence images. NS = not significant, ***p < 0.001, one-way ANOVA followed by Tukey's multiple comparisons test, symbols represent each cell, n =7–20 cells per hydrogel. Bars represent mean ± SEM.

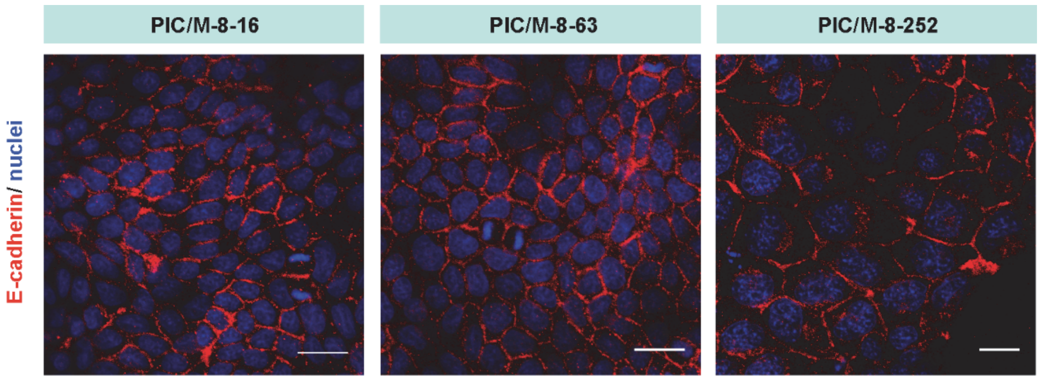

Figure S13. Representative E-cadherin staining images of MDCK cells on PIC/M-8 substrate of different RGD density. All images are representative of n = 3 independent biological experiments. Scale bar: 20 µm.

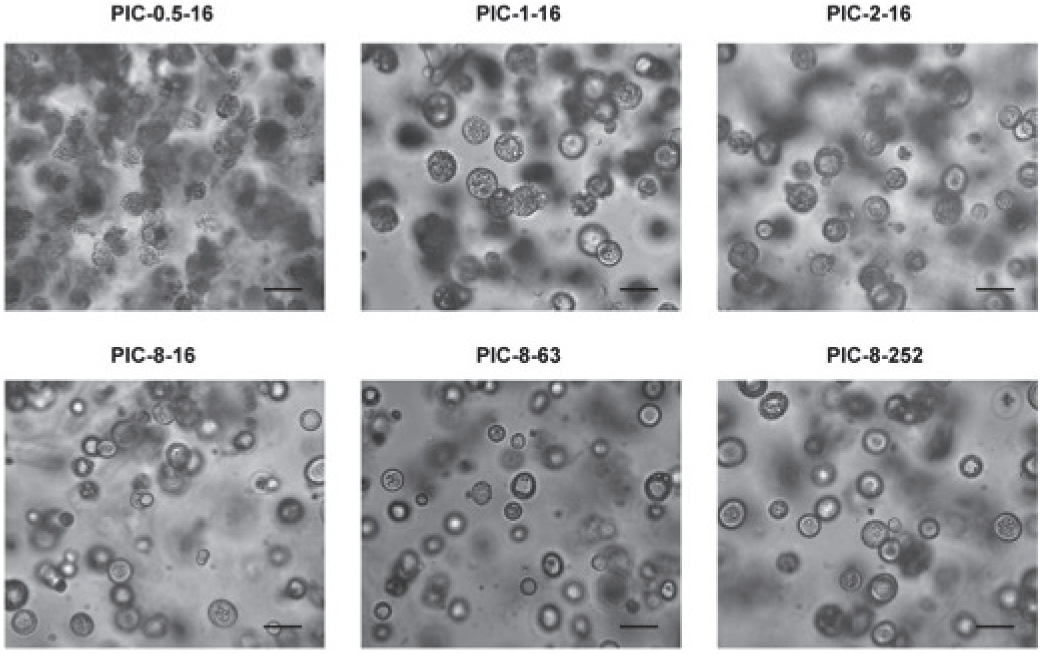

Figure S14. Representative bright field images of MDCK cells in PICs (3D) of different formulations. Scale bar: 100 µm.

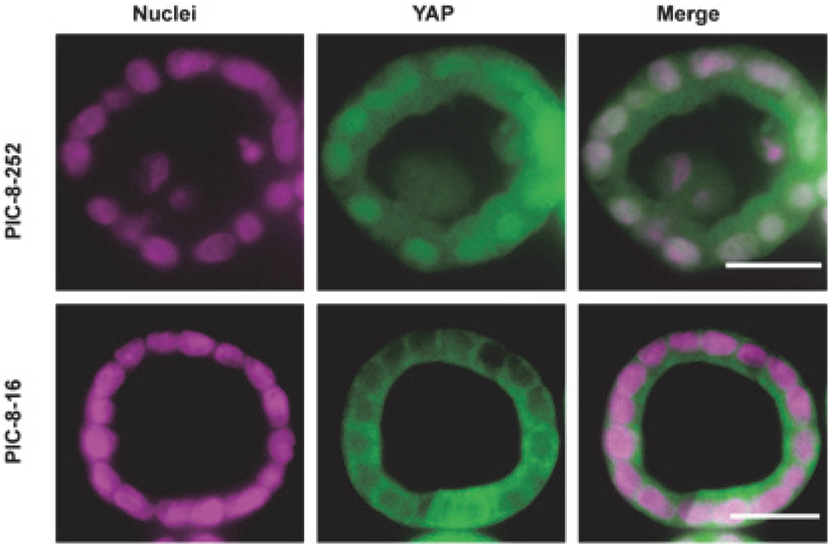

Figure S15. Representative immunofluorescence images of YAP (green) and nuclei (purple) of MDCK cells in PICs of highest stiffness. Scale bar: 20 µm.

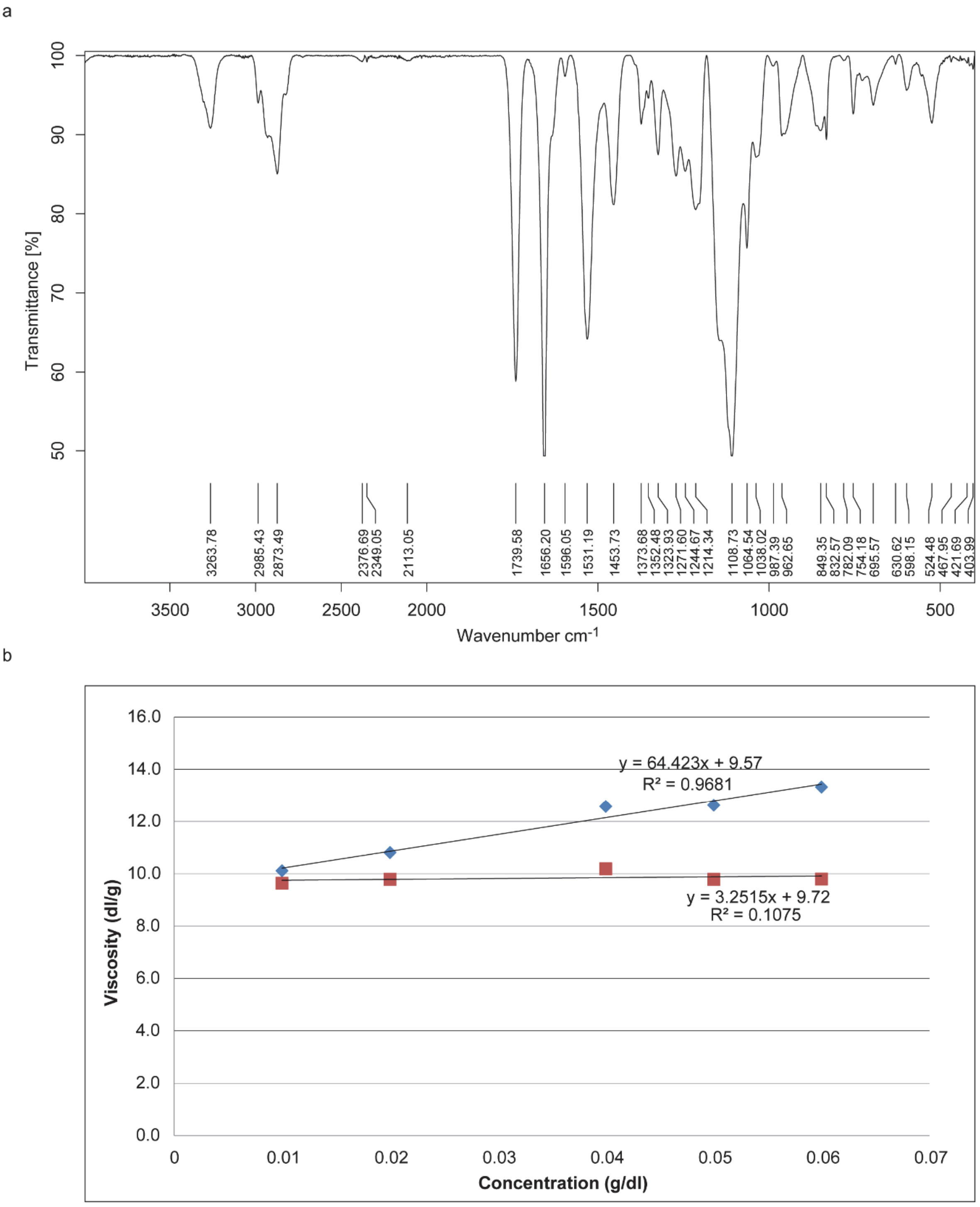

Figure S16. Polymer characterization. (a) IR spectra of PIC polymers. (b) Viscometry analysis of the PIC polymer in acetonitrile solution. The related molecular weight M_v = 419 kg mol^{-1} was calculated based on polymer concentration and its viscosity data on PIC-azide in acetonitrile solutions. Blue triangles: the reduced viscosity, Red triangles: the intrinsic viscosity.

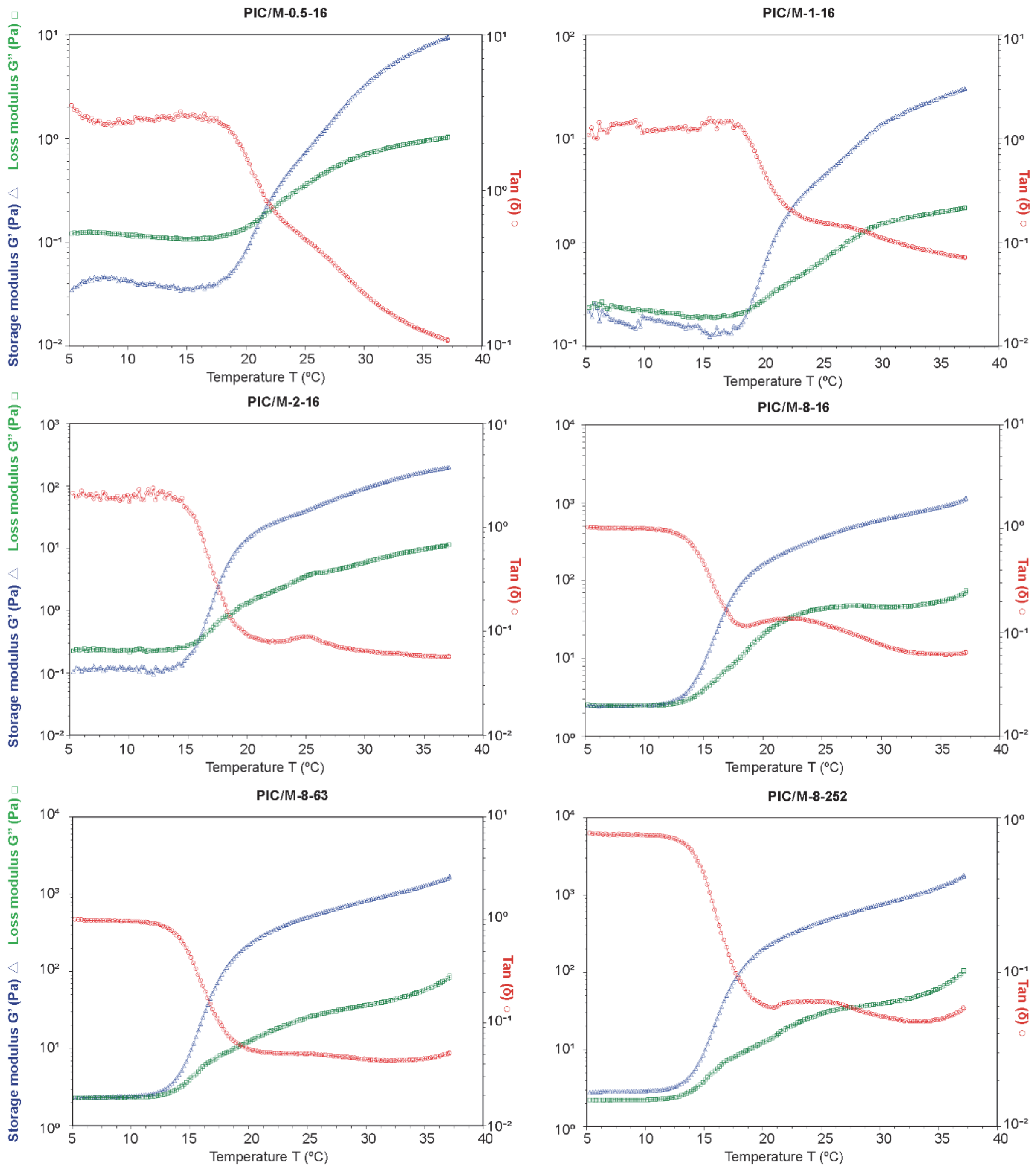

Figure S17. Representative rheology data a heating ramp from 5 °C to 37 °C in HBSS buffer at of PIC/M-0.5-16, PIC/M-1-16 of PIC/M-2-16, PIC/M-8-16, PIC/M-8-63 and PIC/M-8-252. Averages of *G*′ at *T* = 37 °C over 3 independent measurements are given in **Figure S3a**.

Supplementary Movie 1, 2. Representative 3D views, that indicate that both components are tightly mixed.

Supplementary Movie 3. (a) Video shows 3D cysts formation on PIC/M-0.5 with 16 µM RGD conjugated on PIC gels. (b) Video shows 2D monolayer sheets formation on PIC/M-2 with 16 µM RGD conjugated on PIC gels. (c) Structure of 3D cysts generated on PIC/M-0.5 with 16 µM RGD conjugated on PIC gels. Each layer was acquired by z-stack function of confocal and the video was generated by Fiji software.

Table S1. Morphology, polarity and YAP localization in 2D PIC hydrogels and PIC/M composites. Pink background means: control experiments without Matrigel; n.d. = not determined. #Cysts as defined are monolayer round hollow 3D structures.

RGD density on PIC (µM)	Gel	Stiffness	Morphology	polarization apical side faces:	YAP location
0	PIC/M-0.5-0	Soft	Round (but not hollow) clusters or small monolayer clusters	Medium	n.d.
	PIC-0.5-0	Soft	Big cell clusters and clumps	Medium	n.d.
	PIC/M-1-0	Mediate soft	Round (but not hollow) clusters or small monolayer clusters	Medium	n.d.
	PIC-1-0	Mediate soft	Big cell clusters and clumps	Medium	n.d.
	PIC/M-2-0	Mediate soft	Round (but not hollow) clusters or small monolayer clusters	Medium	n.d.
	PIC-2-0	Mediate soft	Big cell clusters and clumps	Medium	n.d.
	PIC/M-8-0	Stiff	Big cell clusters and clumps	Medium	n.d.
	PIC-8-0	Stiff	Big cell clusters and clumps	Medium	n.d.
16	PIC/M-0.5-16	Soft	Hollow cysts#	Lumen	Cytoplasm
	PIC-0.5-16	Soft	Monolayers	Medium	Cytoplasm
	PIC/M-1-16	Mediate soft	Monolayers	Medium	Cytoplasm
	PIC-1-16	Mediate soft	Monolayers	Medium	Cytoplasm
	PIC/M-2-16	Mediate soft	Monolayers	Medium	Cytoplasm
	PIC-2-16	Mediate soft	Monolayers	Medium	Cytoplasm
	PIC/M-8-16	Stiff	Monolayers	Medium	Cytoplasm
	PIC-8-16	Stiff	Monolayers	Medium	Cytoplasm
63	PIC/M-8-63	Stiff	Monolayers	n.d.	Cytoplasm
	PIC-8-63	Stiff	Monolayers	n.d.	Cytoplasm
252	PIC/M-8-252	Stiff	Monolayers	n.d.	Nuclei
	PIC-8-252	Stiff	Monolayers	n.d.	Cytoplasm

Table S2: Morphology, polarity and YAP localization in 3D PIC hydrogels and PIC/M composites. *We defined a multilayer of round cell clusters with little lumen as incomplete cysts.

RGD density on PIC (µM)	Gel	Stiffness	Morphology	polarization apical side faces:	YAP location
0	PIC/M-8-0	Stiff	Incomplete hollow cysts	n.d.	Cytoplasm
	PIC-8-0	Stiff	Solid round cell clusters not hollow	n.d.	n.d.
16	PIC/M-0.5-16	Soft	Incomplete hollow cysts*	n.d.	Cytoplasm
	PIC-0.5-16	Soft	Hollow cyst	n.d.	n.d.
	PIC/M-2-16	Mediate soft	Solid and disorganized cell clusters	n.d.	Cytoplasm
	PIC-2-16	Mediate soft	Hollow cyst	n.d.	n.d.
	PIC/M-8-16	Stiff	Incomplete hollow cysts	n.d.	Cytoplasm
	PIC-8-16	Stiff	Hollow cyst	n.d.	Cytoplasm
252	PIC/M-8-252	Stiff	Tubular structures	n.d.	Nuclei
	PIC-8-252	Stiff	Hollow cyst	n.d.	Nuclei

[1] J. S. Liu, J. T. Farlow, A. K. Paulson, M. A. Labarge, Z. J. Gartner, *Cell Rep.* **2012**, *2*, 1461.
